# Sinapaldehyde Induces Mitochondrial Dysfunction and Modulates Autophagy/Mitophagy-Associated Signaling and Apoptosis in MDA-MB-231 Triple-Negative Breast Cancer Cells

**DOI:** 10.3390/ijms27209359

**Published:** 2026-10-21

**Authors:** Hiruni Sandunika Kumarasinghe, Ducdat Le, Ji-Hyang Kim, Su-Lim Kim, Kyeoung Cheol Kim, Rambukka-na Maggonage Thiruni Dananjana Perera, Yaejun Kim, Mina Lee, Dong-Sun Lee

**Affiliations:** 1 Interdisciplinary Graduate Program in Advanced Convergence Technology & Science, Jeju National University, Jeju 63243, Republic of Korea; hiruni.sandunika@stu.jejunu.ac.kr (H.S.K.); cheolst2@jejunu.ac.kr (K.C.K.); thiruniperera@stu.jejunu.ac.kr (R.-n.M.T.D.P.); yaejunkim98@jejunu.ac.kr (Y.K.); 2 College of Pharmacy and Research Institute of Life and Pharmaceutical Sciences, Sunchon National University, Suncheon, Jeonnam-Gwangju 57922, Republic of Korea; ddle@scnu.ac.kr; 3 Department of Natural Cosmetics Science and AI Digital Bioscience Research Institute, Sunchon National University, Suncheon, Jeonnam-Gwangju 57922, Republic of Korea; 4 AI Bio Autonomous Research Core Facility Center, Sunchon National University, Suncheon, Jeonnam-Gwangju 57922, Republic of Korea; 5 Bio-Health Materials Core-Facility Center, Jeju National University, Jeju 63243, Republic of Korea; seogwi12@jejunu.ac.kr; 6 Graduate Program for Bio-Health/Innovative Drug Development Using Subtropical Bio-Resources, Jeju National University, Jeju 63243, Republic of Korea; ksl1101@jejunu.ac.kr; 7 Faculty of Biotechnology, College of Applied Life Sciences, Jeju National University, Jeju 63243, Republic of Korea

**Keywords:** apoptosis, autophagy, ferritinophagy, mitochondrial dysfunction, sinapaldehyde, network pharmacology

## Abstract

Triple-negative breast cancer (TNBC) is highly aggressive and lacks targeted therapies. This study investigated the anti-cancer mechanisms of sinapaldehyde (SNAH). The biological effects of SNAH were evaluated in vitro using MDA-MB-231 TNBC cells. Cellular responses, including autophagy, mitochondrial function, apoptosis, cell-cycle progression, migration, and colony formation were investigated. In silico studies explored associated regulatory networks and binding targets. Sinapaldehyde (SNAH) treatment was associated with mitochondrial dysfunction, characterized by alterations in mitochondrial structural and functional markers, increased reactive oxygen species(ROS) production, and loss of mitochondrial membrane potential. SNAH treatment also altered the expression of PINK1 and Parkin and modulated autophagy-associated markers, including LC3B, p62, LAMP1, Beclin-1, and ATG proteins. Time-dependent changes in autophagy- and apoptosis-associated markers were accompanied by increased Annexin V-positive cells and activation of cleaved caspase-9 and caspase-3. SNAH also altered FTH1 and NCOA4 expression, suggesting modulation of ferritinophagy-associated processes, and inhibited migration and colony formation while inducing G2/M cell-cycle arrest. In silico analyses identified potential breast cancer-related molecular targets and signaling pathways associated with SNAH. SNAH induces mitochondrial dysfunction and is associated with alterations in autophagy/mitophagy-associated signaling and activation of apoptosis in MDA-MB-231 TNBC cells. These findings provide insight into the cellular responses to SNAH treatment, while further functional studies are required to establish changes in mitophagic and ferritinophagic flux and the causal relationship between autophagy and apoptosis.

## 1. Introduction

Breast cancer is among the most prevalent malignancies and the leading cause of cancer-related deaths in women worldwide. Triple-negative breast cancer (TNBC) is distinguished from other subtypes by the absence of estrogen receptor (ER), progesterone receptor (PR), and human epidermal growth factor receptor 2 (HER2) expression [1,2]. TNBC accounts for approximately 1.7 million women out of all breast cancer cases and is characterized by high rates of metastasis, aggressive clinical progression, limited treatment options, and poor prognosis [3]. Unlike hormone receptor-positive or HER2-positive breast tumors, TNBC lacks well-defined molecular targets; therefore, conventional chemotherapy remains a primary systemic treatment option [4]. However, chemotherapy resistance and treatment-associated toxicity severely limit favorable patient outcomes. Chemotherapy resistance in TNBC is multifactorial and can involve increased drug efflux, enhanced DNA damage repair, evasion of apoptosis, epithelial–mesenchymal transition (EMT), cancer stem-cell enrichment, metabolic adaptation, and activation of pro-survival signaling pathways [5]. These mechanisms can enable TNBC cells to survive chemotherapy and contribute to disease recurrence, highlighting the critical need for innovative and effective therapeutic strategies for TNBC.

Natural products are rich sources of anti-cancer agents with novel scaffolds and mechanisms of action [6]. Sinapaldehyde, a naturally occurring phenolic aldehyde found in various plants, has recently garnered attention owing to its anti-inflammatory and anti-cancer properties [7]. It is primarily derived from the oxidative degradation of lignin, a complex aromatic polymer that forms the major structural component of plant cell walls [8]. Pistacia vera (pistachio) byproducts, particularly pistachio shells, are desirable lignocellulose resources [9]. Pistachio shells contain approximately 23–24% lignin, 24–25% cellulose, and 37–38% hemicellulose by dry weight [10]. Notably, lignin extracted from pistachio shells contains syringyl (S) unit monolignol precursors closely related to sinapaldehyde and sinapyl alcohol, making this biomass a promising feedstock for sinapaldehyde production [11]. Oxidative or enzymatic depolymerization of S-unit-rich biomass can produce aromatic chemicals such as syringaldehyde and sinapaldehyde [12]. Considering the abundance of pistachio shells (PSs) (constituting > 50% of the fruit mass in many cultivars) and their rich content of S-lignin, they form a viable starting point for sinapaldehyde extraction via targeted biomass processing. Thus, pistachio shells demonstrate potential as agricultural waste as well as lignocellulosic feedstock with intrinsic biochemical relevance for extracting phenolic aldehydes, including sinapaldehyde [10]. However, the presence of sinapaldehyde in pistachio shell lignin breakdown products remains to be directly confirmed experimentally. Further, the molecular mechanisms underlying the anti-cancer effects of sinapaldehyde, particularly in triple-negative breast cancer (TNBC), remain unclear.

Mitochondrial dynamics and quality control are critical regulators of cellular homeostasis, energy metabolism, and fate. In cancer, mitochondrial dysfunction is intricately linked to tumor progression, metabolic reprogramming, and resistance to apoptosis [13]. Mitophagy, a selective form of autophagy that targets damaged or dysfunctional mitochondria, is vital to maintaining mitochondrial integrity and cell survival [8]. The PINK1-Parkin pathway is the most extensively studied mitophagy mechanism, wherein PINK1 accumulation in damaged mitochondria recruits Parkin to initiate its degradation via autophagosomes. Mitophagy dysregulation has been implicated in cancer development and therapeutic resistance, making it an attractive target for anti-cancer interventions [8]. Considering the critical roles of mitochondrial dysfunction and autophagy regulation in cancer cell survival, examining the effects of sinapaldehyde on these pathways may uncover new therapeutic opportunities. Therefore, in this study, we investigated the effects of sinapaldehyde in MDA-MB-231 TNBC cells, with particular focus on mitochondrial function, autophagy/mitophagy-associated signaling, oxidative stress, apoptosis, and related cellular responses.

## 2. Results

### 2.1. Molecular Network Guide Identification of SNAH from PS Extract

**Figure 1:**
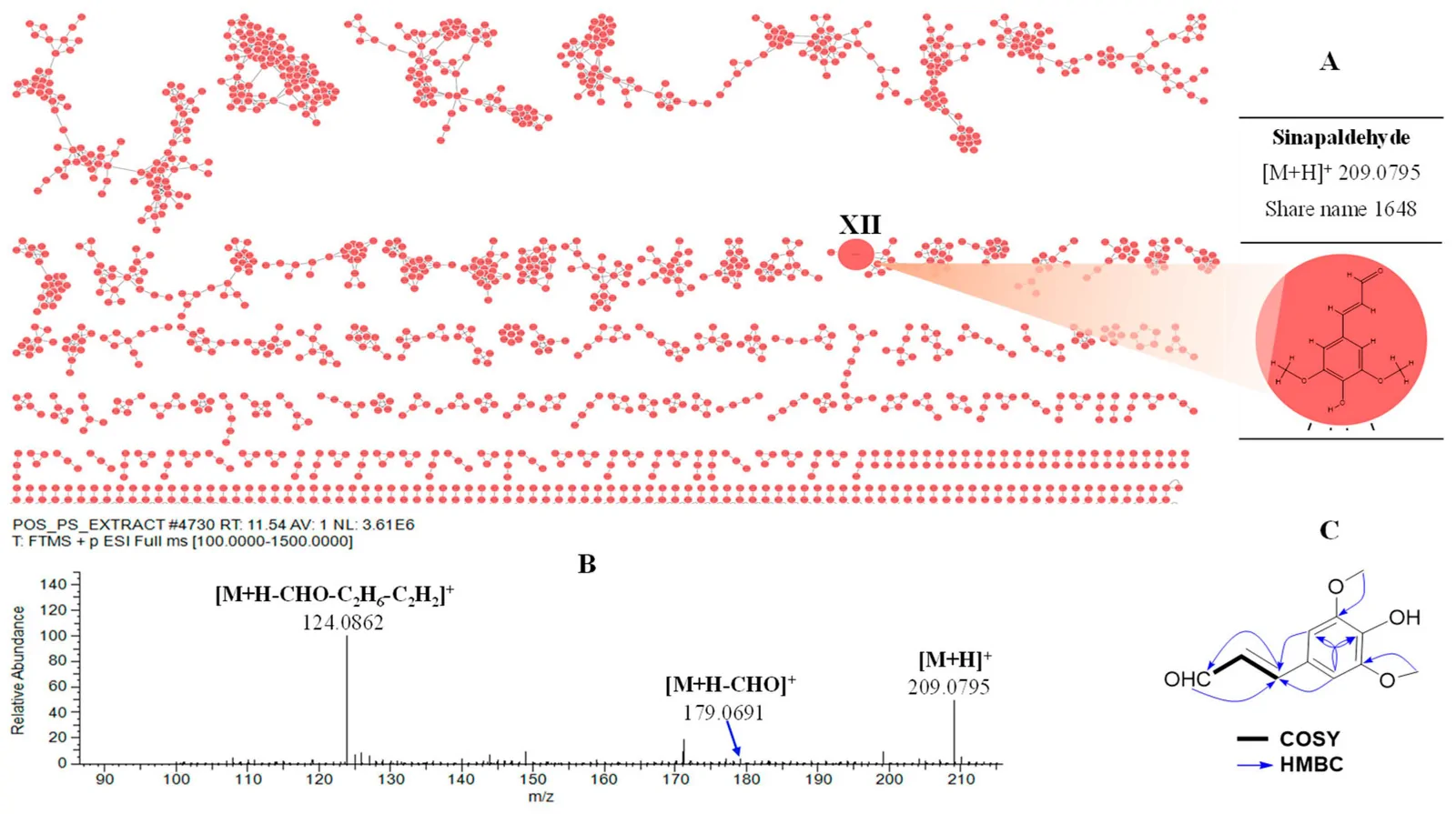
(**A**) Molecular network identification of SNAH. (**B**) HR-ESI-MS/MS data of SNAH peak. (**C**) Key 2D-NMR correlations of SNAH.

The GNPS platform established a feature-based molecular network using high-resolution tandem mass data from the PS extract to identify the presence of SNAH. Therefore, the SNAH compound was precisely and efficiently isolated from sub-fraction E2 of the E fraction from the PS extract. Cluster XII showed a peak node (*t*_R_ 10.8 min, *m*/*z* 209.0795 in positive ion mode) (Figure 1A), which was annotated by using online GNPS tools. The HR-ESI-MS/MS spectrum of this peak displayed a protonated ion peak at *m*/*z* 209.0795 [M+H]^+^ (calculated for C_11_H_12_O_4_H, 209.0814). At the same time, mass fragmentation showed the ion peaks at *m*/*z* 179.0691 [M+H-CHO]^+^ and at *m*/*z* 124.0862 [M+H-CHO-C_2_H_6_]^+^, suggesting the reduction of an aldehyde and two methyl groups, respectively (Figure 1B and Figure S1 in Supplementary Materials). Subsequently, the PS extract was separated by using multiple chromatographic techniques to produce compound RT10.8 (Figure S2–S8, Supplementary Materials). The ^1^H NMR spectrum of compound RT10.8 showed a singlet at *δ*_H_ 9.58 (1H, s, H-3′), a *trans*-olefinic proton system at *δ*_H_ 7.59 (1H, d, *J* = 15.7 Hz, H-1′) and 6.69 (1H, d, *J* = 15.7 Hz, H-2′), two singlets at *δ*_H_ 7.00 (2H, s, H-2/6), and two singlet methoxy groups at *δ*_H_ 3.90 (6H, s, H_3_-3/5). The ^13^C NMR spectrum of RT10.8 exhibited 11 signals, including a carboxylic carbon at *δ*_C_ 196.1 (C-3′), four quaternary carbons at *δ*_C_ 149.6 (C-3/5), 140.8 (C-4), and 126.5 (C-1), four methines at *δ*_C_ 156.2 (C-1′), 126.8 (C-2′), and 107.3 (C-2/6), and two methoxy groups at *δ*_C_ 56.9 (OCH_3_-3/5). The heteronuclear multiple bond correlation (HMBC) spectrum particularly demonstrated the correlations from H-2/H-6 (*δ*_H_ 7.00) to C-4 (*δ*_C_ 140.8)/C-1′ (*δ*_C_ 126.8), while the HMBC correlations of H-1′ (*δ*_H_ 7.59) were observed with C-2/C-6 (*δ*_C_ 107.3)/C-3′ (*δ*_C_ 196.1), indicating that the acryl aldehyde moiety is attached to C-1 of the phenyl substitute. Additionally, HMBC long-range correlation from two methoxy groups at *δ*_H_ 3.90 to C-3/C-5 (*δ*_C_ 149.6) confirmed that the O-CH_3_ functional groups are connected to the C-3 and C-5 positions (Figure 1C). With the above observations, the structure of RT10.8 was identified as sinapaldehyde [14].

### 2.2. SNAH Inhibits the Proliferation of MDA-MB-231 Cancer Cells

**Figure 2:**
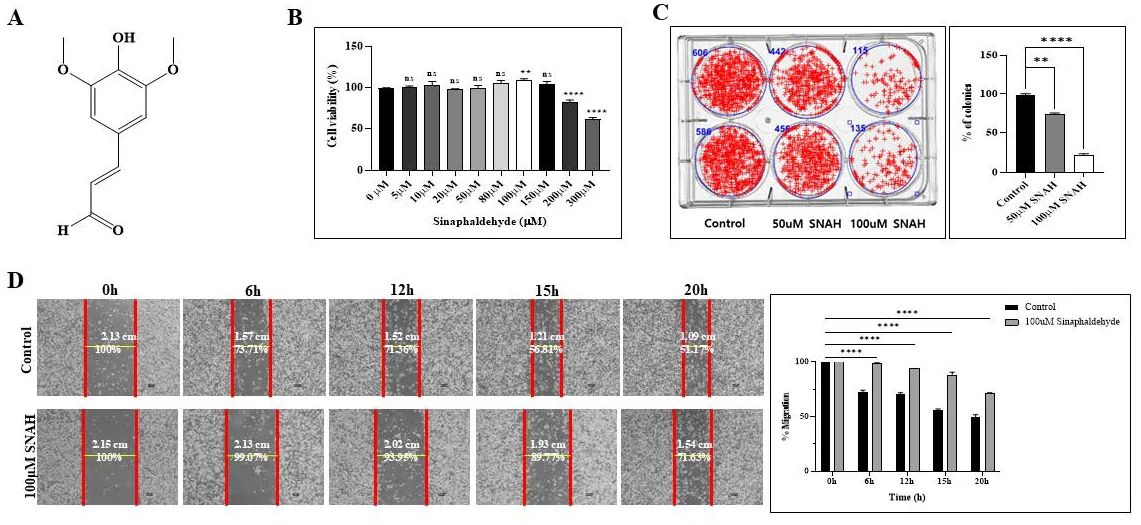

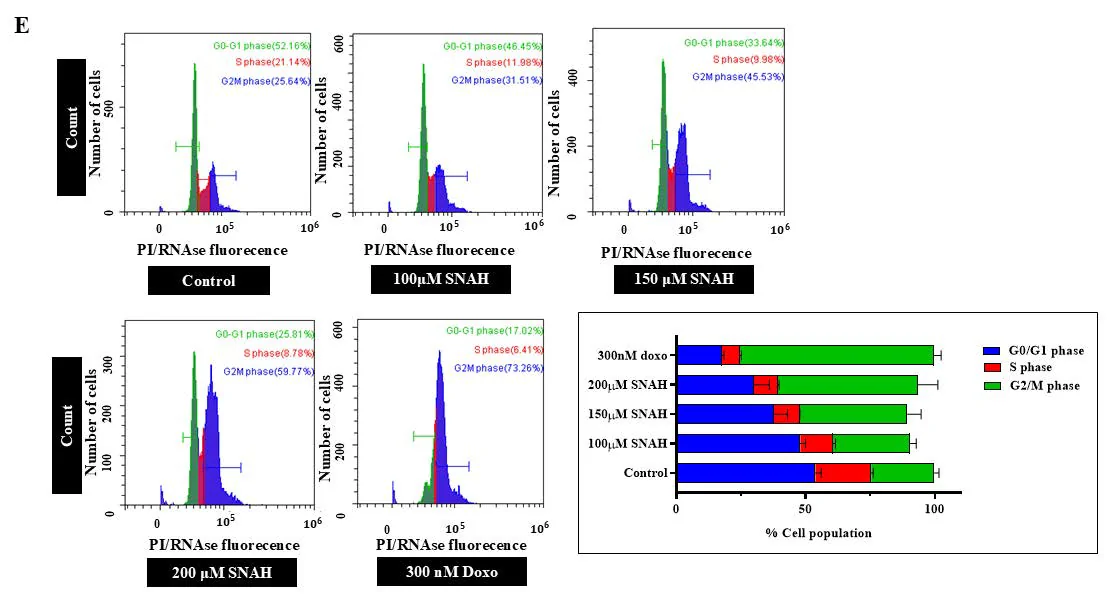
Sinapaldehyde reduces the proliferation of MDA-MB-231 cancer cells. (**A**) The chemical structure of sinapaldehyde. (**B**) The proliferation of cancer cells was assessed with WST reagent after 24 h SNAH treatment. (**C**) The inhibitory effect of 50 μM and 100 μM sinapaldehyde on colony formation. Cells were cultured in a plate with the sinapaldehyde for 24 h and colonies were stained using crystal violet to check the colony formation. (**D**) Cell migration assay of MDA-MB-231 cells upon sinapaldehyde treatment (100 µM). The MDA-MB-231 cells without drug treatment were used as controls. (**E**) Cell cycle detection after treating MDA-MB-231 cells with 100 μM, 150 μM and 200 μM SNAH or 300 nM doxorubicin (positive control) for 24 h. Data are presented as mean ± SD from three independent biological replicates (n = 3). Statistical significance for the cell viability, colony formation, and cell-cycle assays was analyzed using one-way ANOVA followed by Tukey’s multiple-comparisons test. The wound-healing assay was analyzed using two-way repeated-measures ANOVA followed by a multiple-comparisons test. ** *p* < 0.01 and **** *p* < 0.0001.

To explore the antiproliferative effects of sinapaldehyde (SNAH) on MDA-MB-231 breast cancer cells, a series of functional assays were performed. Colony formation assays revealed a dose-dependent reduction in colony numbers upon treatment with 50 µM and 100 µM SNAH, indicating impaired long-term proliferative capacity. The quantification revealed a significant decrease in colony formation compared with that in the untreated control group (*p* < 0.0001) (Figure 2A–C). Furthermore, a scratch wound healing assay demonstrated that SNAH at 100 µM significantly suppressed cell migration over 24 h, with reduced wound closure percentages at 6, 12, 15, and 20 h (*p* < 0.0001), suggesting strong anti-migratory potential (Figure 2D).

Flow cytometry was performed to determine whether the observed growth inhibition was linked to cell cycle modulation. SNAH treatment resulted in a marked accumulation of cells in the G2/M phase, rising from 25.64% in the control to 31.51%, 45.53%, and 59.77% in the 100 µM, 150 µM, and 200 µM SNAH groups, respectively.

This was accompanied by a corresponding reduction in the G0/G1 and S phases, suggesting that SNAH induced G2/M cell cycle arrest. These effects were comparable to those seen with 300 nM doxorubicin. Together, these findings suggest that SNAH effectively inhibits TNBC cell proliferation by suppressing colony formation, reducing cell migration, and inducing G2/M phase arrest, potentially by activating mitochondria-mediated apoptotic pathways (Figure 2E).

### 2.3. SNAH Induces Cell Death in MDA-MB-231 Cells via Apoptosis

**Figure 3:**
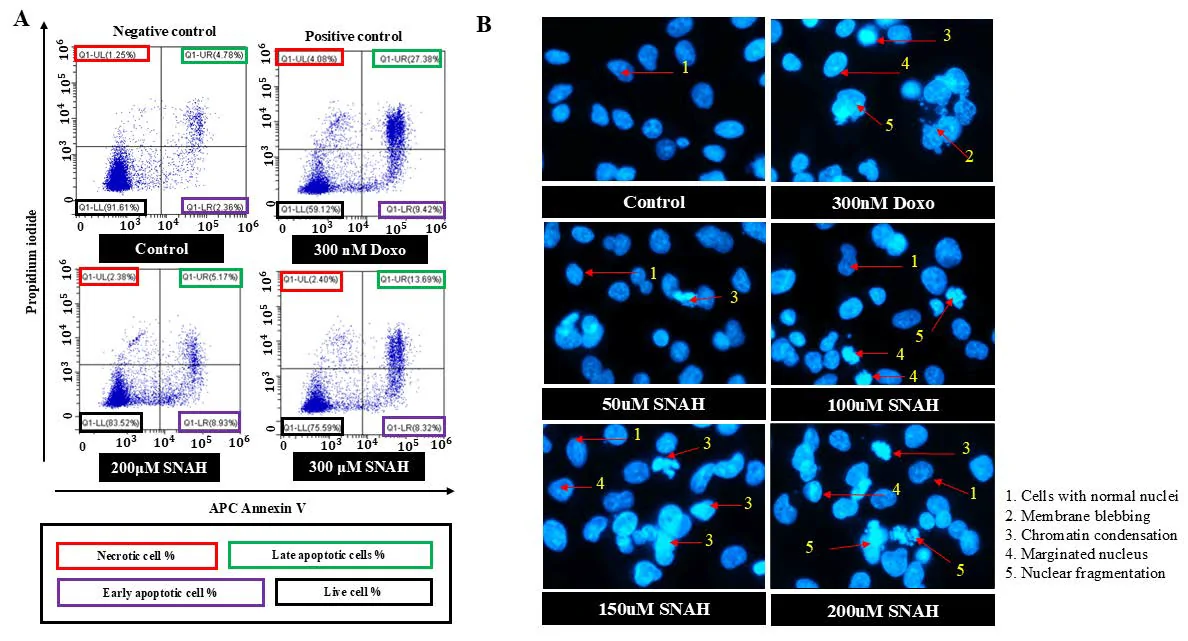
MDA-MB-231 cell apoptosis was analyzed by flow cytometry after Annexin-V APC/PI staining and Hoechst staining. (**A**) Cells were treated with 200 and 300 μM SNAH for 24 h and apoptosis was checked via Annexin V-APC/PI staining. The 300 nM doxorubicin was used as the control and media only was used as the negative control. (**B**) Morphological changes visualized by fluorescence microscopy after Hoechst staining.

To determine whether SNAH inhibited MDA-MB-231 cell proliferation through apoptosis induction, Annexin V-APC/PI flow cytometry analysis and nuclear morphology assessment using Hoechst staining were performed. As shown in Figure 3A, flow cytometric analysis revealed that the untreated control group predominantly comprised viable cells, whereas treatment with the positive control 300 nM doxorubicin, led to a substantial increase in the late apoptotic and necrotic cell populations. SNAH treatment at 200 and 300 µM resulted in a progressive and dose-dependent increase in both the early and late apoptotic populations, indicating effective apoptotic induction by SNAH (Figure 3A).

Consistent with the flow cytometry results, Hoechst 33342 nuclear staining (Figure 3B) confirmed apoptosis through distinct morphological changes. Control cells showed uniform, round nuclei, whereas cells treated with doxorubicin or SNAH (50–200 µM) showed classical apoptotic features such as chromatin condensation, nuclear margination, fragmentation, and membrane blebbing. These nuclear alterations became more prominent with increasing SNAH concentration, supporting the dose-dependent nature of its cytotoxic effects. Overall, these findings demonstrate that SNAH induces apoptosis in MDA-MB-231 cells, contributing to its anti-proliferative activity.

### 2.4. SNAH Inhibits the MDA-MB-231 Cancer Cell Proliferation by Inducing Mitochondrial Apoptosis

**Figure 4:**
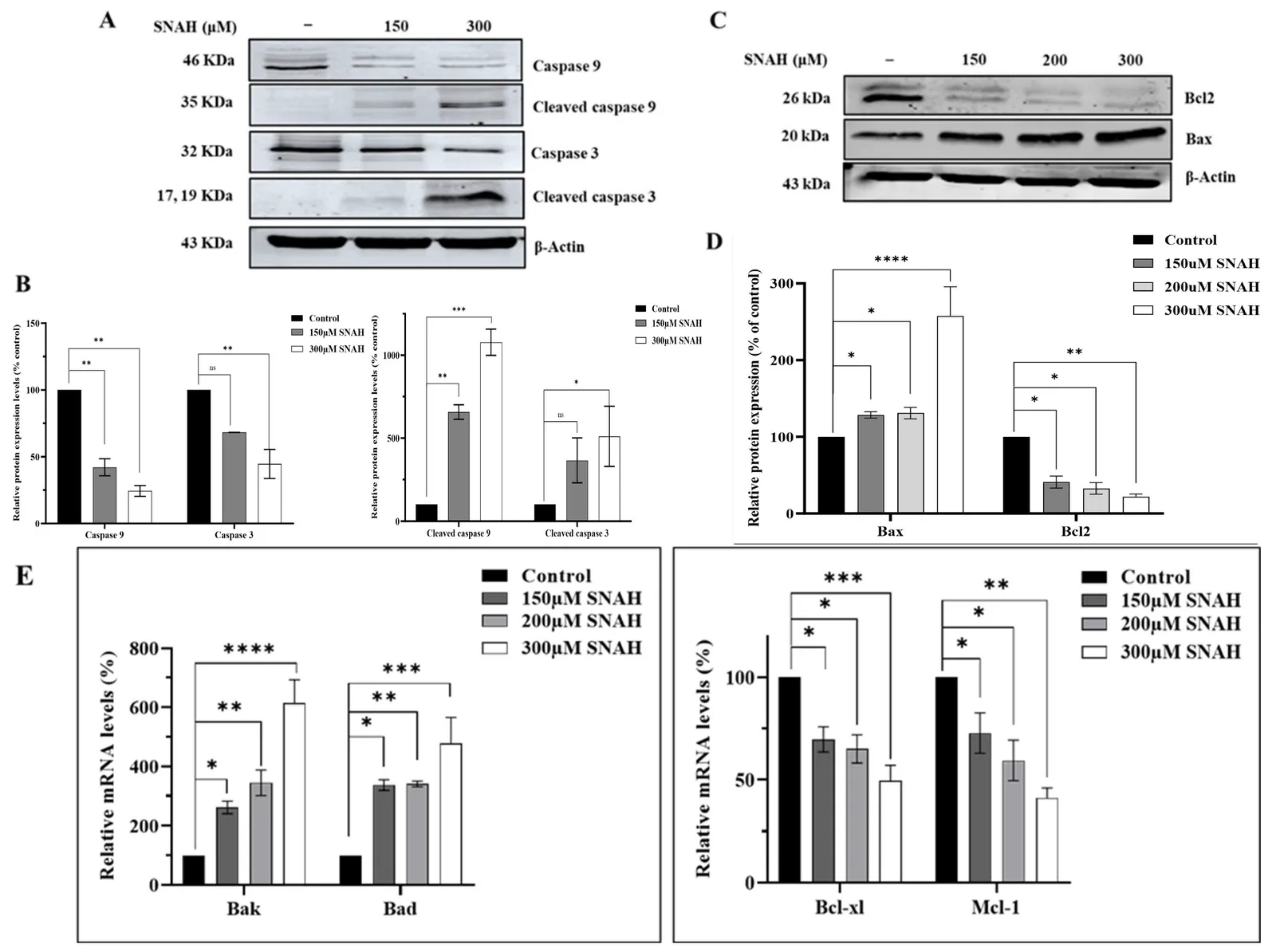

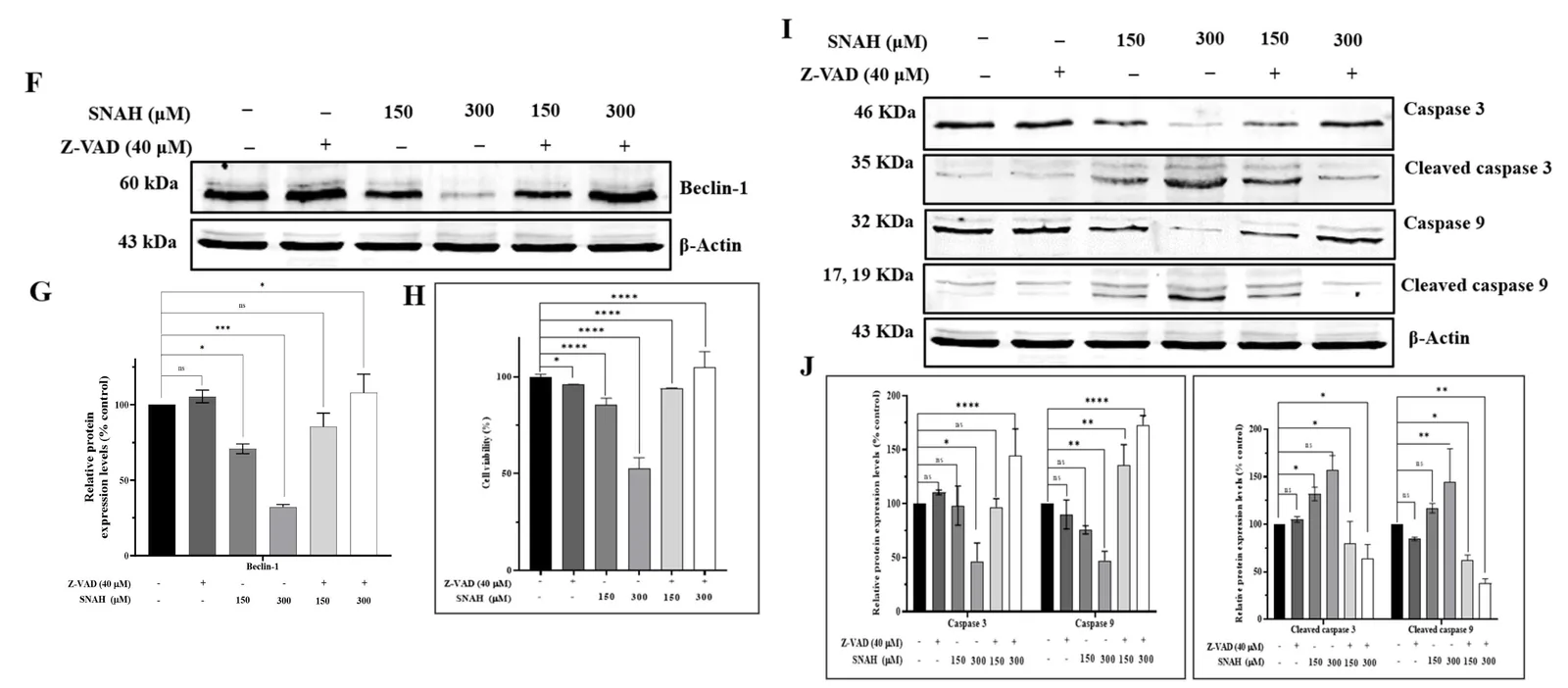
Effect of SNAH treatment on apoptosis-related proteins in MDA-MB-231 cells. (**A**) Western blot analysis of caspase-3, cleaved caspase-3, caspase-9, and cleaved caspase-9 in cells treated with 150 µM and 300 µM SNAH. (**B**) β-Actin was used as a loading control and densitometric analysis of protein bands, normalized to β-actin, showed relative protein expression levels compared to the control. (**C**) Western blot analysis of Bax and Bcl-2 in cells treated with 150 µM and 300 µM SNAH. (**D**) β-Actin was used as a loading control and densitometric analysis of protein bands, normalized to β-actin, showed relative protein expression levels compared to the control. (**E**) qPCR analysis of the pro-apoptotic and anti-apoptotic markers after treating with 150 μM, 200 μM and 300 μM of sinapaldehyde for 24 h. (**F**) MDA-MB-231 cells were treated with SNAH for 24 h in the presence or absence of 40 μM Z-VAD-FMK (pan-caspase inhibitor) and Beclin-1 expression was determined by Western blot. (**G**) Densitometric analysis of protein bands, normalized to β-actin, showing relative protein expression levels compared to the control. (**H**) Cell viability was determined by WST assay following SNAH treatment in the presence or absence of Z-VAD-FMK (40 μM). (**I**) MDA-MB-231 cells were treated with SNAH for 24 h in the presence or absence of 40 μM Z-VAD-FMK (pan-caspase inhibitor) and caspase expression was determined by Western blot. (**J**) Densitometric analysis of protein bands, normalized to β-actin, showing relative protein expression levels compared to the control. Data are presented as mean ± SD from three independent biological replicates (n = 3). Statistical significance for SNAH concentration-dependent experiments (**B**,**D**,**E**) was analyzed using one-way ANOVA followed by Tukey’s multiple-comparisons test. For experiments involving SNAH and Z-VAD-FMK co-treatment (**G**,**H**,**J**), two-way ANOVA followed by Tukey’s multiple-comparisons test was used. * *p* < 0.05, ** *p* < 0.01, *** *p* < 0.001 and **** *p* < 0.0001.

To further confirm activation of the mitochondrial apoptotic pathway by SNAH, Western blotting and qPCR analyses were performed to assess the expression of key apoptotic regulators. As shown in Figure 4A, Western blotting revealed a marked increase in the cleaved (active) forms of caspase-9 and caspase-3 following treatment with 150 µM and 300 µM SNAH. This activation of initiator and executioner caspases strongly indicated induction of intrinsic (mitochondria-mediated) apoptosis (Figure 4A,B).

Additionally, the expression of Bcl-2 family proteins was evaluated (Figure 4C,D). SNAH treatment led to a concentration-dependent increase in proapoptotic Bax expression and a concurrent decrease in anti-apoptotic Bcl-2 levels, resulting in an elevated Bax/Bcl-2 ratio. At the transcriptional level (Figure 4E), SNAH significantly upregulated the mRNA levels of the pro-apoptotic genes Bak and Bad, whereas it downregulated the anti-apoptotic genes Bcl-xL and Mcl-1. These findings further support the involvement of mitochondrial apoptotic signaling in the response to SNAH treatment.

To functionally validate the contribution of caspase-dependent apoptosis to SNAH-induced cell death, MDA-MB-231 cells were treated with SNAH in the presence or absence of the pan-caspase inhibitor Z-VAD-FMK (40 µM). As shown in Figure 4H, Z-VAD-FMK co-treatment markedly attenuated the SNAH-induced reduction in cell viability, with the rescue being particularly evident following treatment with 300 µM SNAH. This finding indicates that caspase activity contributes functionally to SNAH-induced cytotoxicity. Consistent with the viability results, Z-VAD-FMK co-treatment reduced the SNAH-induced increase in cleaved caspase-3 and cleaved caspase-9 levels (Figure 4I,J), further supporting the involvement of caspase-dependent apoptotic signaling. In addition, Z-VAD-FMK co-treatment altered Beclin-1 expression compared with SNAH treatment alone (Figure 4F,G), suggesting an association between caspase-dependent apoptosis and the observed changes in autophagy-associated signaling. Collectively, these results provide functional evidence that caspase-dependent apoptosis contributes to SNAH-induced cell death in MDA-MB-231 cells.

### 2.5. SNAH Exhibits Modest Antioxidant Activity Compared to That of Standard Antioxidants

**Figure 5:**
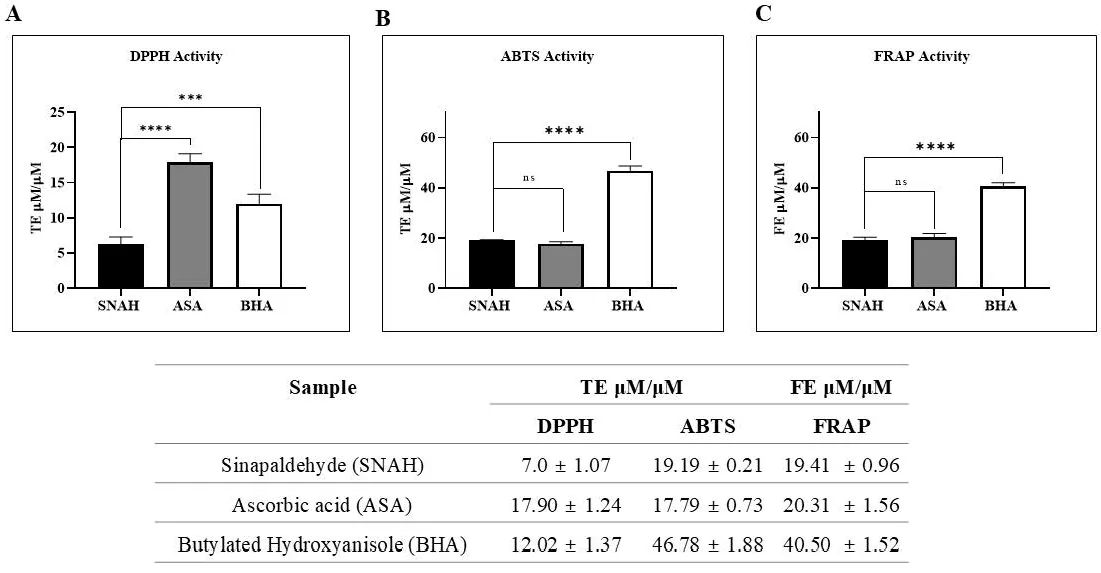
Antioxidant activity of sinapaldehyde (SNAH). (**A**) DPPH (2,2-diphenyl-1-picrylhydrazyl) radical scavenging activity of SNAH compared to ascorbic acid (ASA) and butylated hydroxyanisole (BHA) as positive controls. (**B**) ABTS (2,2′-azino-bis(3-ethylbenzothiazoline-6-sulfonic acid)) radical scavenging activity of SNAH in comparison to ASA and BHA. (**C**) Ferric reducing antioxidant power (FRAP) assay showing the reducing capacity of SNAH relative to ASA and BHA. Data are presented as means ± SD. Data are presented as mean ± SD from three independent biological replicates (n = 3). Statistical significance was analyzed using one-way ANOVA followed by Tukey’s multiple-comparisons test. *** *p* < 0.001 and **** *p* < 0.0001.

To assess the antioxidant potential of SNAH, three in vitro radical scavenging assays, DPPH, ABTS, and FRAP, were conducted and the results were compared with those of standard antioxidants. As shown in Figure 5A, the DPPH assay revealed that SNAH possessed significantly lower radical scavenging activity (7.0 TE µM/µM) compared with both ASA (17.90 TE µM/µM) and BHA (12.02 TE µM/µM). Similarly, in the ABTS assay (Figure 5B), SNAH demonstrated a radical scavenging capacity of 19.19 TE µM/µM, which was markedly lower than that of BHA (46.78 TE µM/µM), but comparable to that of ASA (17.79 TE µM/µM).

Consistent with these findings, the FRAP assay (Figure 5C) indicated that SNAH showed lower ferric reducing antioxidant power (19.41 FE µM/µM) compared to that of BHA (40.50 FE µM/µM), and a similar level of activity as that of ASA (20.31 FE µM/µM). Collectively, these results demonstrate that although SNAH possesses antioxidant capacity, its radical scavenging and reducing power are modest and significantly lower than those of standard antioxidants. This suggests that the pro-apoptotic and anti-cancer effects of SNAH in MDA-MB-231 cells are likely independent of its antioxidant properties.

### 2.6. SNAH Modulates Autophagy- and Mitophagy-Associated Markers in MDA-MB-231 Cells

**Figure 6:**
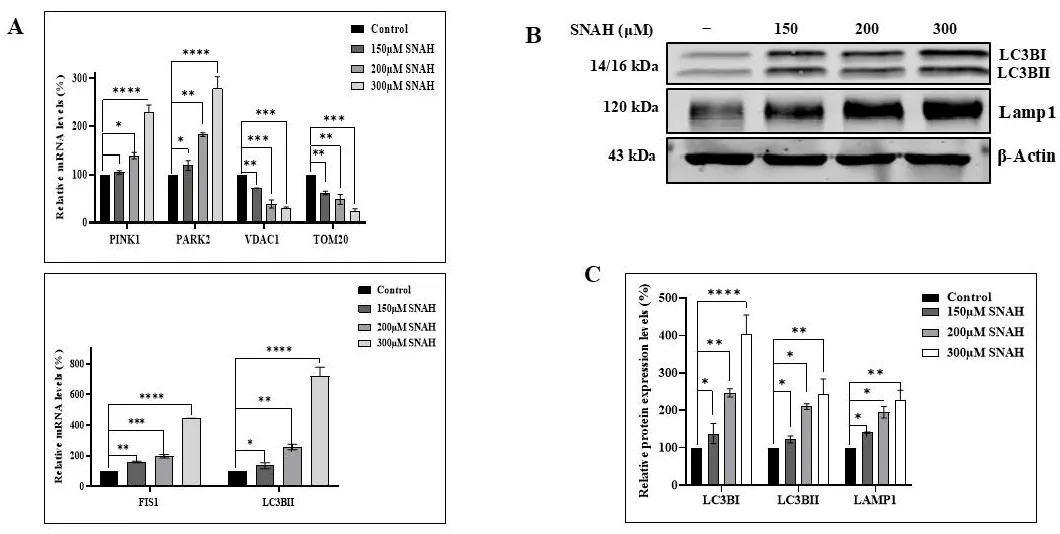
Sinapaldehyde treatment alters autophagy- and mitophagy-associated markers in MDA-MB-231 cells. (**A**) After treatment of 150, 200, and 300 μM of sinapaldehyde, PINK1, Parkin, VDAC1, TOM20, FIS1 and LC3B2 mRNA expression levels were analyzed by qPCR. (**B**) Autophagy-related protein expression level of MDA-MB-231 cells treated with sinapaldehyde for 24 h analyzed by Western blotting. (**C**) Densitometric analysis of protein bands, normalized to β-actin, showing relative protein expression levels compared to the control. Data are presented as mean ± SD from three independent biological replicates (n = 3). Statistical significance was analyzed using one-way ANOVA followed by Tukey’s multiple-comparisons test. * *p* < 0.05, ** *p* < 0.01, *** *p* < 0.001 and **** *p* < 0.0001.

To examine whether SNAH alters mitophagy-associated markers, Western blot analysis was performed to evaluate the key markers of mitochondrial dynamics and autophagy. As shown in Figure 6A, SNAH treatment altered the mRNA expression of PINK1 and Parkin, indicating transcriptional modulation of these mitophagy-associated genes. However, changes in PINK1 and Parkin transcript levels do not by themselves establish activation of functional PINK1/Parkin-dependent mitophagy. Further, changes in mitochondrial structural markers such as VDAC1 and TOM20 as well as the mitochondrial fission protein FIS1 imply that SNAH may influence mitochondrial dynamics. The increased LC3B-II levels were consistent with increased autophagosome accumulation; however, LC3B-II abundance alone does not establish increased mitophagic flux.

**Figure 7:**
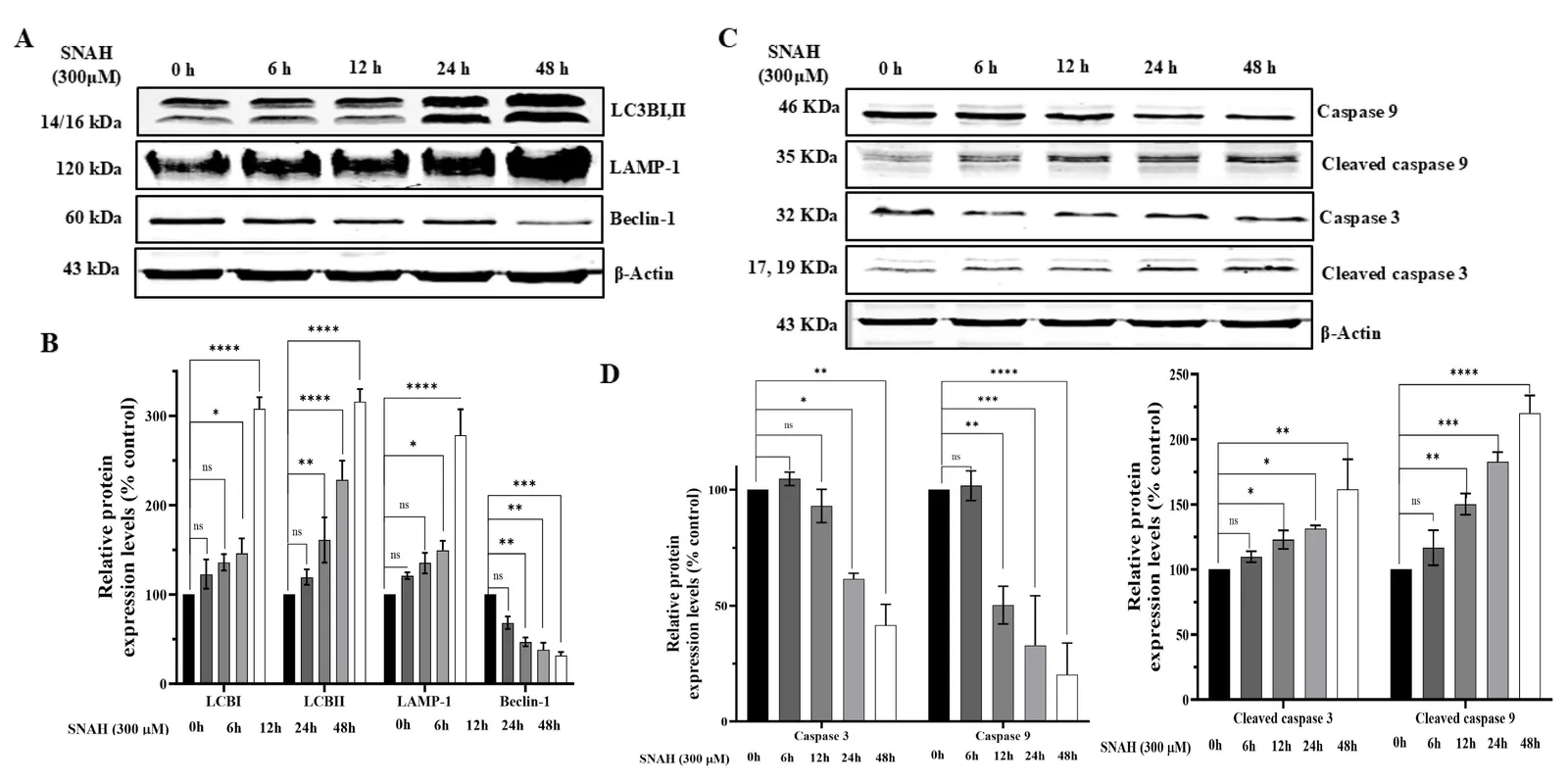
Time-dependent changes in autophagy- and apoptosis-associated markers following SNAH treatment. MDA-MB-231 cells were treated with SNAH (300 µM) for 0–48 h, and autophagy- and apoptosis-related proteins were analyzed in the same experimental batch. (**A**) LC3-II, p62, LAMP-1, and Beclin-1 expression were monitored to characterize time-dependent changes in autophagy-associated markers. LC3-II and LAMP-1 gradually increased up to 24 h, indicating increased abundance of these autophagy- and lysosome-associated markers, followed by a decline in Beclin-1 expression. (**B**) Densitometric analysis of protein bands, normalized to β-actin. (**C**) Cleaved caspase-9 and caspase-3 increased at 24–48 h, indicating activation of the apoptotic pathway. β-Actin was used as the loading control. These results demonstrate a temporal association between changes in autophagy-associated markers and subsequent activation of apoptotic pathways. Because these measurements represent steady-state protein abundance, they do not by themselves establish increased autophagic flux. (**D**) Densitometric analysis of protein bands, normalized to β-actin. Data are presented as mean ± SD from three independent biological replicates (n = 3). Statistical significance among different time points was analyzed using one-way ANOVA followed by Dunnett’s multiple-comparisons test, using 0 h as the control. * *p* < 0.05, ** *p* < 0.01, *** *p* < 0.001 and **** *p* < 0.0001.

Figure 6B,C show changes in autophagy- and lysosome-associated markers following SNAH treatment. SNAH treatment resulted in a pronounced increase in LC3B-II levels, consistent with increased autophagosome accumulation. Moreover, the expression of LAMP1, a lysosomal membrane marker, increased, whereas that of p62 decreased. The reduction in p62 together with increased LC3B-II and LAMP1 levels indicates modulation of the autophagy–lysosome system. However, these steady-state changes alone are insufficient to establish increased autophagic flux. Collectively, these findings indicate that SNAH alters autophagy- and mitophagy-associated markers; however, direct assessment of mitophagic flux and PINK1/Parkin pathway activity is required to determine whether these changes reflect functional activation or inhibition of mitophagy. Further, according to Figure 7A,B, Western blot analysis revealed that SNAH (300 μM) treatment significantly increased LC3B-II and LAMP-1 expression, while Beclin-1 expression gradually decreased over 48 h, indicating time-dependent changes in autophagy-associated markers. Figure 7C,D show the cleaved caspase-3 and cleaved caspase-9 increased markedly in a time-dependent manner, accompanied by a reduction in the total caspase-3 and caspase-9 levels, suggesting the induction of mitochondria-mediated apoptosis. Densitometric analysis confirmed these changes, showing significant differences compared with the untreated control group.

### 2.7. SNAH Alters Mitochondrial Membrane Potential and PINK1/Parkin-Associated Proteins

**Figure 8:**
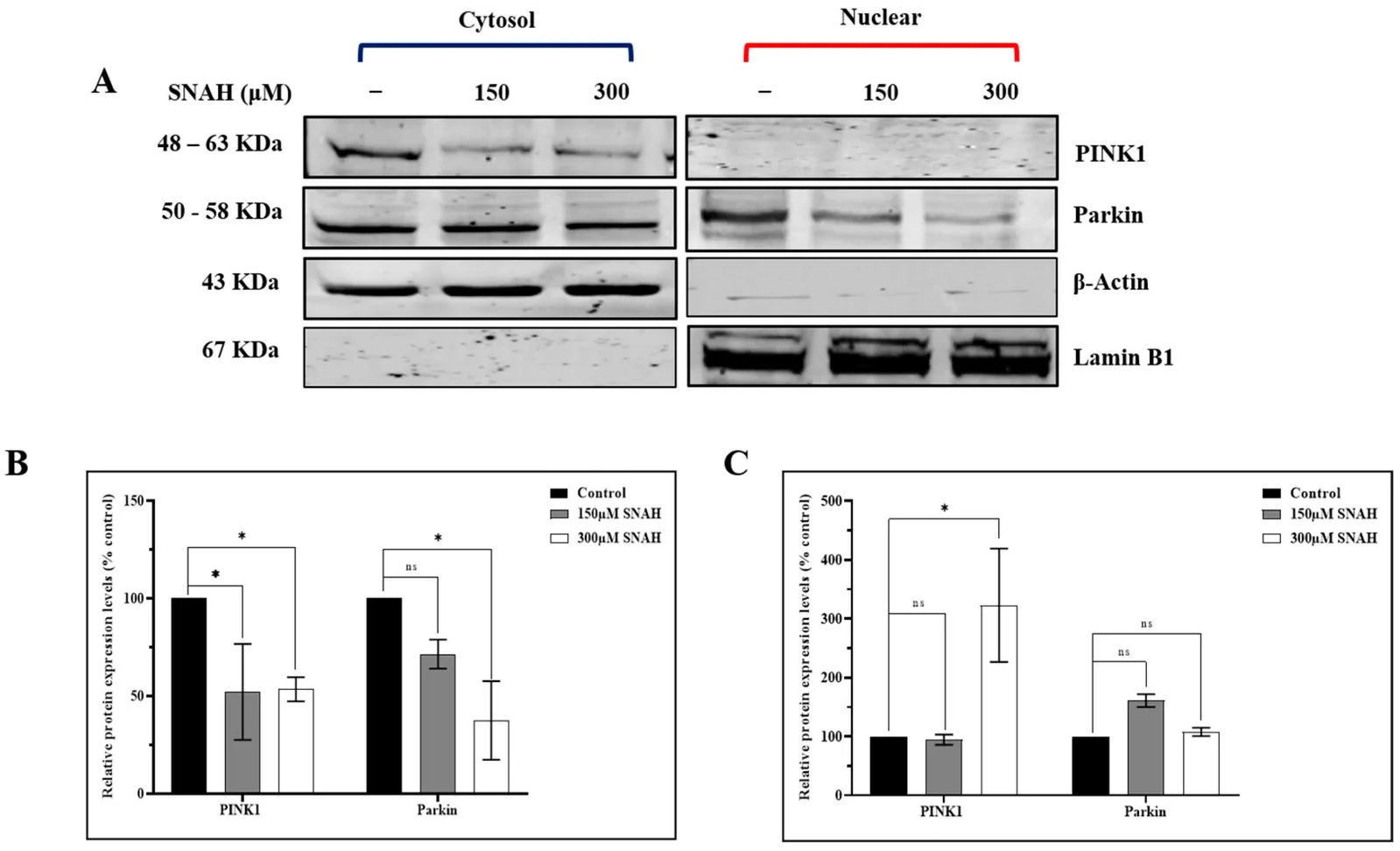
SNAH alters PINK1/Parkin-associated protein expression in MDA-MB-231 cells. (**A**) Western blot analysis of cytosolic and nuclear PINK1, and Parkin in cells treated with 150 µM and 300 µM SNAH. β-Actin was used as a loading control for the cytosolic fraction and lamin B1 was used as the loading control for the nuclear fraction. (**B**) Densitometric analysis of protein bands, normalized to β-actin for the cytosolic protein fraction, showing relative protein expression levels compared to the control. (**C**) Densitometric analysis of protein bands, normalized to lamin B1 for the nuclear protein fraction, showing relative protein expression levels compared to the control. Data are presented as mean ± SD from three independent biological replicates (n = 3). Statistical significance was analyzed using one-way ANOVA followed by Tukey’s multiple-comparisons test. * *p* < 0.05.

To further examine PINK1/Parkin-associated responses following SNAH treatment, the subcellular distribution of PINK1 and Parkin was evaluated in cytosolic and nuclear fractions. In contrast to the changes observed at the transcript level, PINK1 and Parkin protein levels were reduced following SNAH treatment (Figure 8A–C). These findings indicate differential regulation of PINK1 and Parkin at the transcript and protein levels. However, changes in steady-state protein abundance do not by themselves establish functional activation or inhibition of PINK1/Parkin-dependent mitophagy [15].

The observed mitochondrial dysfunction was accompanied by alterations in PINK1/Parkin-associated proteins; however, whether these changes result in functional impairment of mitochondrial quality control requires direct assessment of mitophagic flux. These findings support an association between altered PINK1/Parkin-associated mitophagy signaling, mitochondrial stress, and cell death [16].

### 2.8. SNAH Promotes Mitochondrial Dysfunction by Enhancing Fission and Suppressing Fusion Dynamics

**Figure 9:**
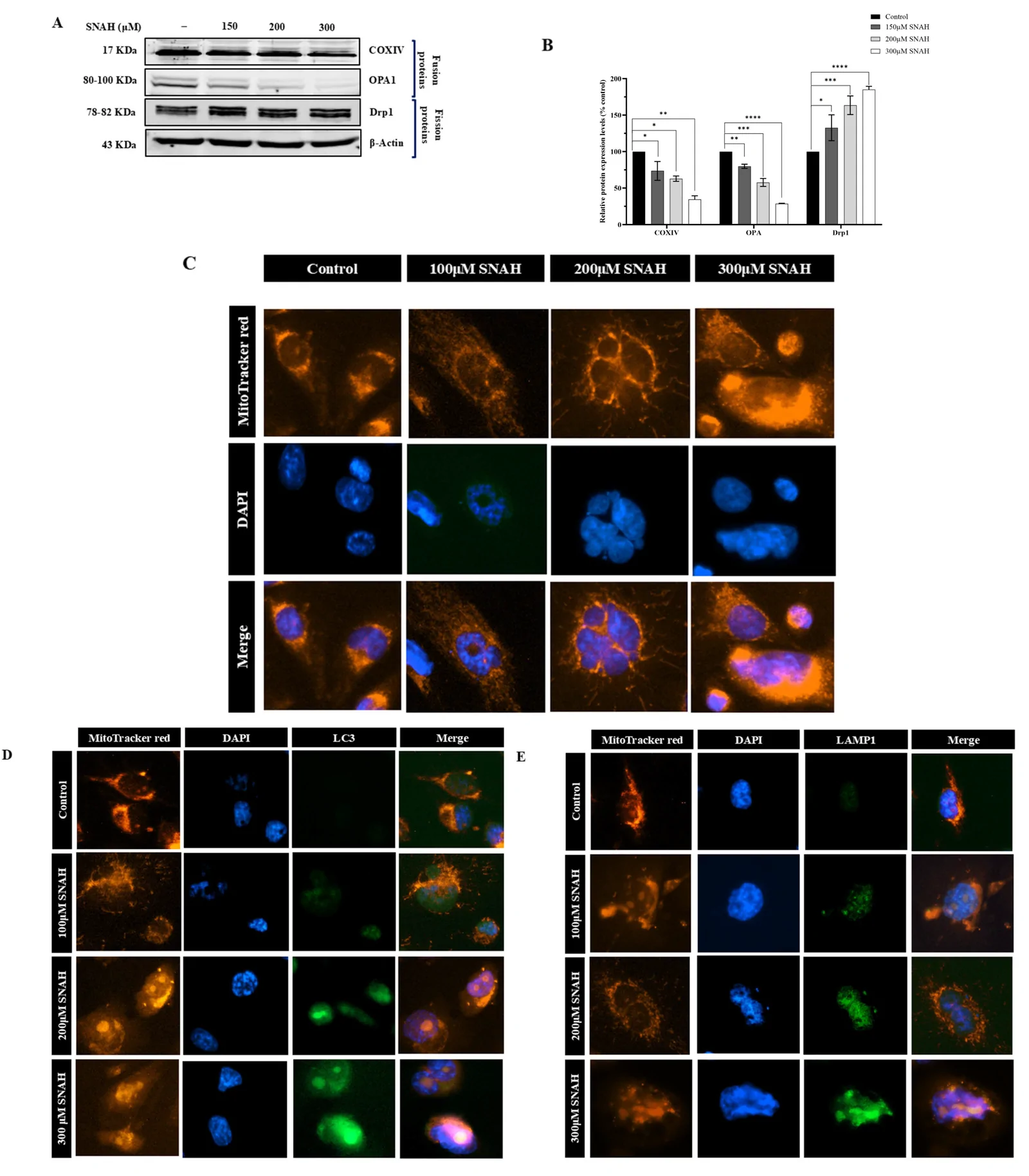
SNAH treatment induces mitochondrial dysfunction in MDA-MB-231 cells. (**A**) Western blot analysis of the protein associated with mitochondrial dynamics (fission and fusion) in cells treated with 150 µM, 200 µM and 300 µM SNAH. β-Actin was used as a loading control. (**B**) Densitometric analysis of protein bands, normalized to β-actin, showing relative protein expression levels compared to the control. (**C**) MDA-MB-231 cells were treated with SNAH for 24 h and then stained with MitoTracker Red (100 nM). The fluorescence was visualized using a fluorescence microscope. Magnification: 20×; scale bar: 100 μm. (**D**,**E**) After treatment with SNAH using different concentrations for 24 h, MDA-MB-231 cells were stained with MitoTracker Red (100 nM) and immunostained with an LC3 or LAMP1 antibody. Mitochondrial colocalization with LC3 or LAMP1 was visualized using a fluorescence microscope. Magnification: 20×; scale bar: 100 μm. Densitometric data are presented as mean ± SD from three independent biological replicates (n = 3). Statistical significance for densitometric analysis was evaluated using one-way ANOVA followed by Tukey’s multiple-comparisons test. * *p* < 0.05, ** *p* < 0.01, *** *p* < 0.001 and **** *p* < 0.0001.

To assess the impact of SNAH on mitochondrial structural integrity, the expression of key regulators of mitochondrial dynamics was analyzed using Western blotting. As shown in Figure 9A,B increasing concentrations of SNAH (150–300 µM) led to a dose-dependent decrease in mitochondrial fusion protein OPA1. Simultaneously, a marked reduction was observed in COXIV, a mitochondrial marker, suggesting overall mitochondrial destabilization. In contrast, expression of the mitochondrial fission protein Drp1 was elevated in response to SNAH treatment.

Figure 9B quantifies these changes, confirming the significant downregulation of fusion-related proteins and relative upregulation of Drp1 after treating with 200 µM and 300 µM SNAH. This imbalance between fusion and fission indicates a shift toward excessive mitochondrial fragmentation. Disruption of mitochondrial dynamics by SNAH may compromise mitochondrial function and integrity, contributing to loss of membrane potential and altered mitophagy-associated responses in MDA-MB-231 cells. To evaluate the effect of sinapaldehyde (SNAH) on mitochondrial integrity and mitophagy, MDA-MB-231 cells were treated with varying concentrations of SNAH for 24 h, followed by MitoTracker Red staining and immunofluorescence labeling for LC3 (an autophagosome marker) and LAMP1 (a lysosomal marker). As shown in the microscopic images (Figure 9C–E), SNAH treatment induced a concentration-dependent decrease in MitoTracker Red fluorescence intensity, indicating mitochondrial depolarization and dysfunction. Furthermore, colocalization analysis revealed an increased overlap between mitochondrial signals and LC3 or LAMP1. These findings indicate increased association of mitochondria with LC3-positive autophagy-associated structures and LAMP1-positive lysosome-associated compartments. These findings further support an association between mitochondrial destabilization and SNAH-induced cell death. Taken together, these findings demonstrate that SNAH triggers mitochondrial stress and alters the association of mitochondria with autophagy- and lysosome-associated compartments. However, direct mitophagic flux assays would be required to establish whether mitochondrial delivery to lysosomes results in functional mitophagic degradation.

### 2.9. Pharmacological Modulation of Mitochondrial Fission and Autophagy Alters SNAH-Induced Cytotoxicity

**Figure 10:**
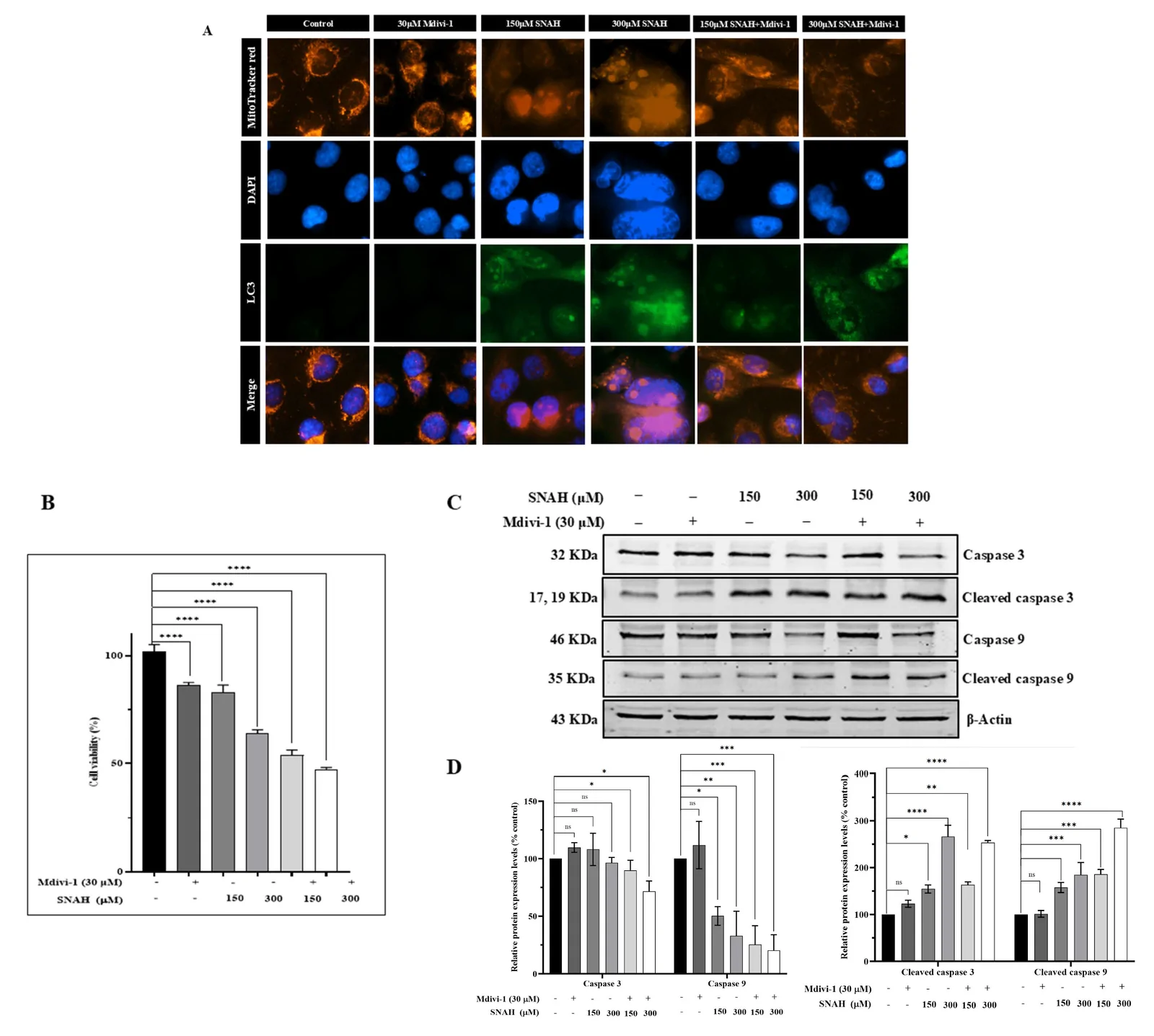

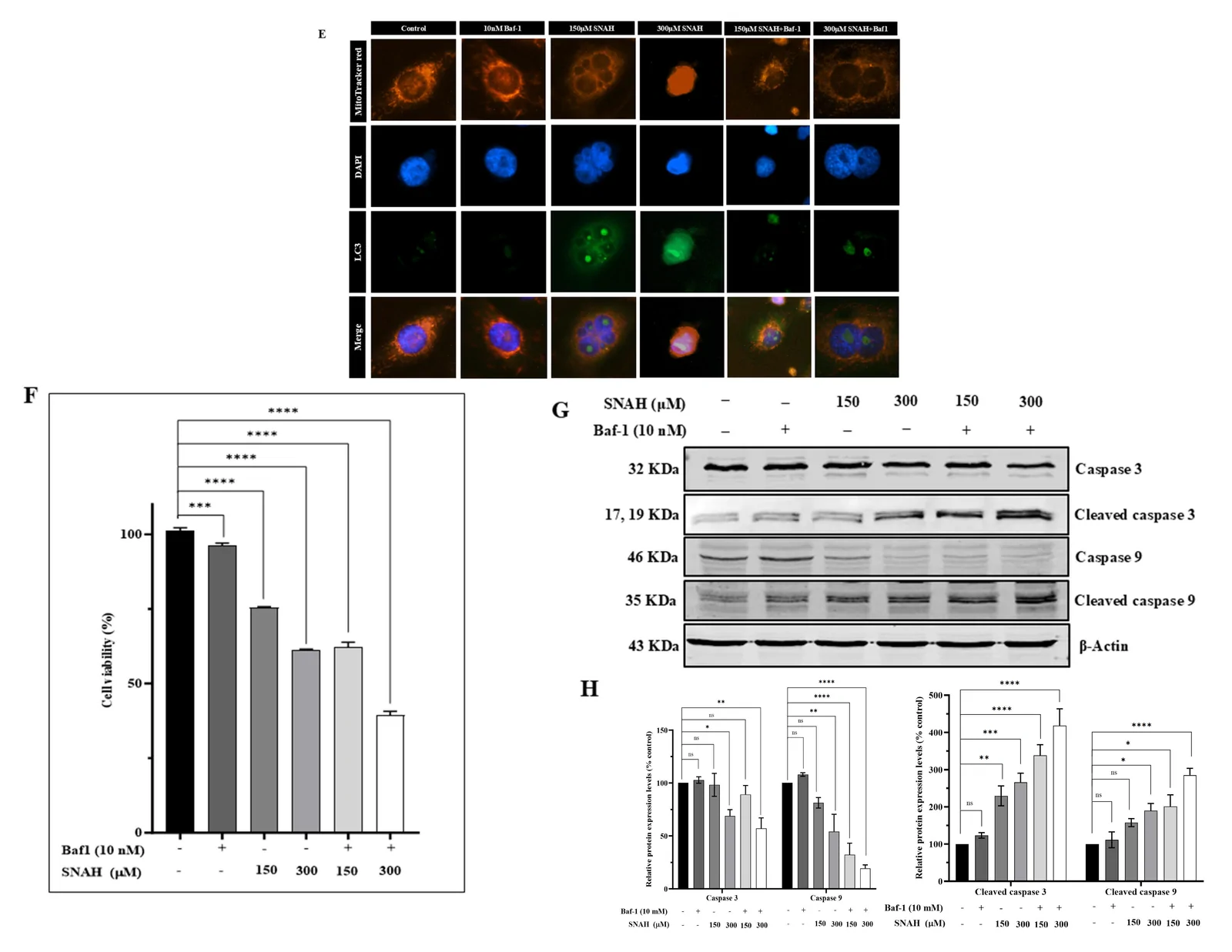
Pharmacological modulation of mitochondrial fission and lysosomal degradation alters SNAH-induced cytotoxicity in MDA-MB-231 cells. (**A**) After treatment with SNAH with Mdivi-1 (30 uM) using different concentrations for 24 h, MDA-MB-231 cells were stained with MitoTracker Red (100 nM) and immunostained with LC3 antibody. (**B**) MDA-MB-231 cells treated with SNAH for 24 h in the presence or absence of 30 uM Mdivi-1 and proliferation activity determined by WST. (**C**) MDA-MB-231 cells treated with SNAH for 24 h in the presence or absence of 30 uM Mdivi-1 and caspase expression determined by Western blot. (**D**) Densitometric analysis of protein bands, normalized to β-actin, showing relative protein expression levels compared to the control. (**E**) After treatment with SNAH with Bafilomycin A1 using different concentrations for 24 h, MDA-MB-231 cells were stained with MitoTracker Red (100 nM) and immunostained with LC3 antibody. (**F**) MDA-MB-231 cells treated with SNAH for 24 h in the presence or absence of 10 nM Bafilomycin A1 and proliferation activity determined by WST. (**G**) MDA-MB-231 cells treated with SNAH for 24 h in the presence or absence of 10 nM Baf-1 and caspase expression determined by Western blot. (**H**) Densitometric analysis of protein bands, normalized to β-actin, showing relative protein expression levels compared to the control. Data are presented as mean ± SD from three independent biological replicates (n = 3). For the co-treatment experiment with Mdivi-1 and Bafilomycin A1, statistical significance was analyzed using two-way ANOVA followed by Tukey’s multiple-comparisons test. * *p* < 0.05, ** *p* < 0.01, *** *p* < 0.001 and **** *p* < 0.0001.

The pharmacological inhibitors Mdivi-1 (mitochondrial fission inhibitor) and Bafilomycin A1 (autophagy inhibitor) were employed to delineate the functional roles of mitochondrial fission and autophagy in SNAH-induced cytotoxicity. Immunofluorescence imaging (Figure 10A) showed that SNAH treatment was associated with mitochondrial fragmentation and LC3 puncta accumulation. Cotreatment with Mdivi-1 reduced these changes, indicating that pharmacological inhibition of mitochondrial fission modifies SNAH-associated mitochondrial and autophagy-related responses [17].

Cell-viability assays (Figure 10B) revealed that Mdivi-1 co-treatment significantly enhanced the SNAH-induced loss of cell viability. However, Western blot analysis (Figure 10C,D) showed that Mdivi-1 co-treatment attenuated SNAH-induced cleavage of caspase-3 and caspase-9. Therefore, the additional loss of viability observed with Mdivi-1 cannot be interpreted as enhanced caspase-dependent apoptosis. These findings indicate that pharmacological inhibition of mitochondrial fission modifies the cellular response to SNAH, although the mechanism underlying the increased cytotoxicity remains unresolved.

To examine the effect of pharmacological inhibition of lysosomal degradation on the cellular response to SNAH, cells were treated with SNAH in the presence or absence of Bafilomycin A1. As shown in Figure 10E, Bafilomycin A1 mitigated SNAH-induced mitochondrial fragmentation and caused marked accumulation of LC3 puncta, consistent with inhibition of lysosomal degradation. Western blotting (Figure 10G,H) revealed that co-treatment with Bafilomycin A1 increased the levels of cleaved caspase-3 and caspase-9, indicating increased apoptosis.

Cell viability assays (Figure 10F) showed that Bafilomycin A1 enhanced SNAH-induced cytotoxicity. Overall, these findings indicate that pharmacological modulation of mitochondrial fission and lysosomal degradation alters the cellular response to SNAH.

### 2.10. SNAH Induces ROS-Mediated Mitochondrial Dysfunction and Apoptosis in MDA-MB-231 Cells

**Figure 11:**
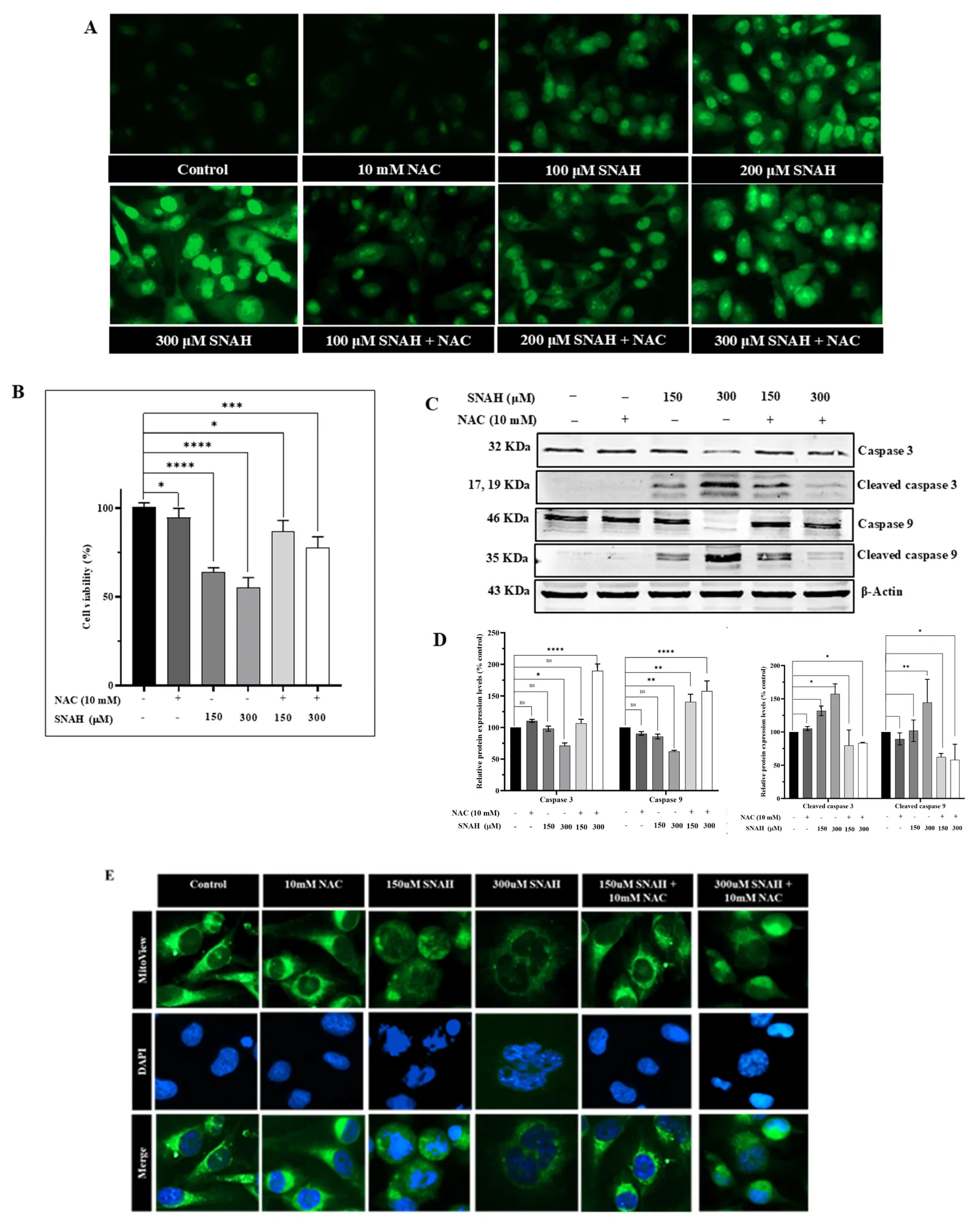
SNAH treatment induces mitochondrial dysfunction and apoptosis associated with ROS overproduction in MDA-MB-231 cells. (**A**) After treatment with sinapaldehyde for 24 h with and without 10 mM NAC, cells were stained with CellROX (5 µM) and visualized under a microscope. Magnification 10×, scale bar 100 µm. (**B**) MDA-MB-231 cells were treated with sinapaldehyde for 24 h in the presence or absence of NAC (10 mM) and cell viability was determined by WST assay. (**C**) MDA-MB-231 cells treated with SNAH for 24 h in the presence or absence of 10 mM NAC and caspase expression determined by Western blot. (**D**) Densitometric analysis of protein bands, normalized to β-actin, showing relative protein expression levels compared to the control. (**E**) After SNAH treatment for the 24 h in the presence or absence of NAC (10 mM), cells were stained with MitoView Green (50 nM) and DAPI, and the mitochondrial morphology was visualized. Magnification 20×, scale bar 100 µm. Quantitative data from the cell viability and densitometric analyses are presented as mean ± SD from three independent biological replicates (n = 3). Statistical significance for experiments involving SNAH and NAC co-treatment was analyzed using two-way ANOVA followed by Tukey’s multiple-comparisons test. * *p* < 0.05, ** *p* < 0.01, *** *p* < 0.001 and **** *p* < 0.0001.

To investigate the involvement of reactive oxygen species (ROS) in SNAH-induced cytotoxicity, intracellular ROS levels were measured using CellROX staining. Treatment with SNAH at increasing concentrations (100 µM, 200 µM, and 300 µM) resulted in a significant, dose-dependent increase in green fluorescence intensity compared to that in control cells, indicating elevated ROS production (Figure 11A). Treatment with the antioxidant N-acetylcysteine (NAC; 10 mM) for 24 h markedly suppressed SNAH-induced ROS generation, confirming the efficacy of NAC in mitigating oxidative stress.

The effect of ROS on cell viability was assessed using a WST assay. SNAH treatment caused a dose-dependent reduction in MDA-MB-231 cell viability (Figure 11B). Notably, NAC pretreatment significantly rescued cells from SNAH-induced cytotoxicity, suggesting that ROS overproduction contributes to SNAH-induced cell death. Consistently, Western blot analysis revealed increased cleavage of caspase-3 and caspase-9 following SNAH exposure, indicating apoptosis induction (Figure 11C,D). NAC pretreatment substantially attenuated caspase cleavage, supporting the involvement of ROS in apoptotic signaling. Mitochondrial integrity was assessed using MitoView Green staining. The control cells showed healthy tubular mitochondrial networks, whereas SNAH treatment induced marked mitochondrial fragmentation, swelling, and depolarization (Figure 11E). NAC pretreatment largely preserved mitochondrial morphology and prevented SNAH-induced mitochondrial damage. Collectively, these results demonstrate that SNAH triggers mitochondrial dysfunction and apoptosis in MDA-MB-231 cells primarily via ROS overproduction and that antioxidant intervention effectively mitigates these deleterious effects.

### 2.11. Sinapaldehyde Induces Mitochondrial Membrane Depolarization in MDA-MB-231 Cells

**Figure 12:**
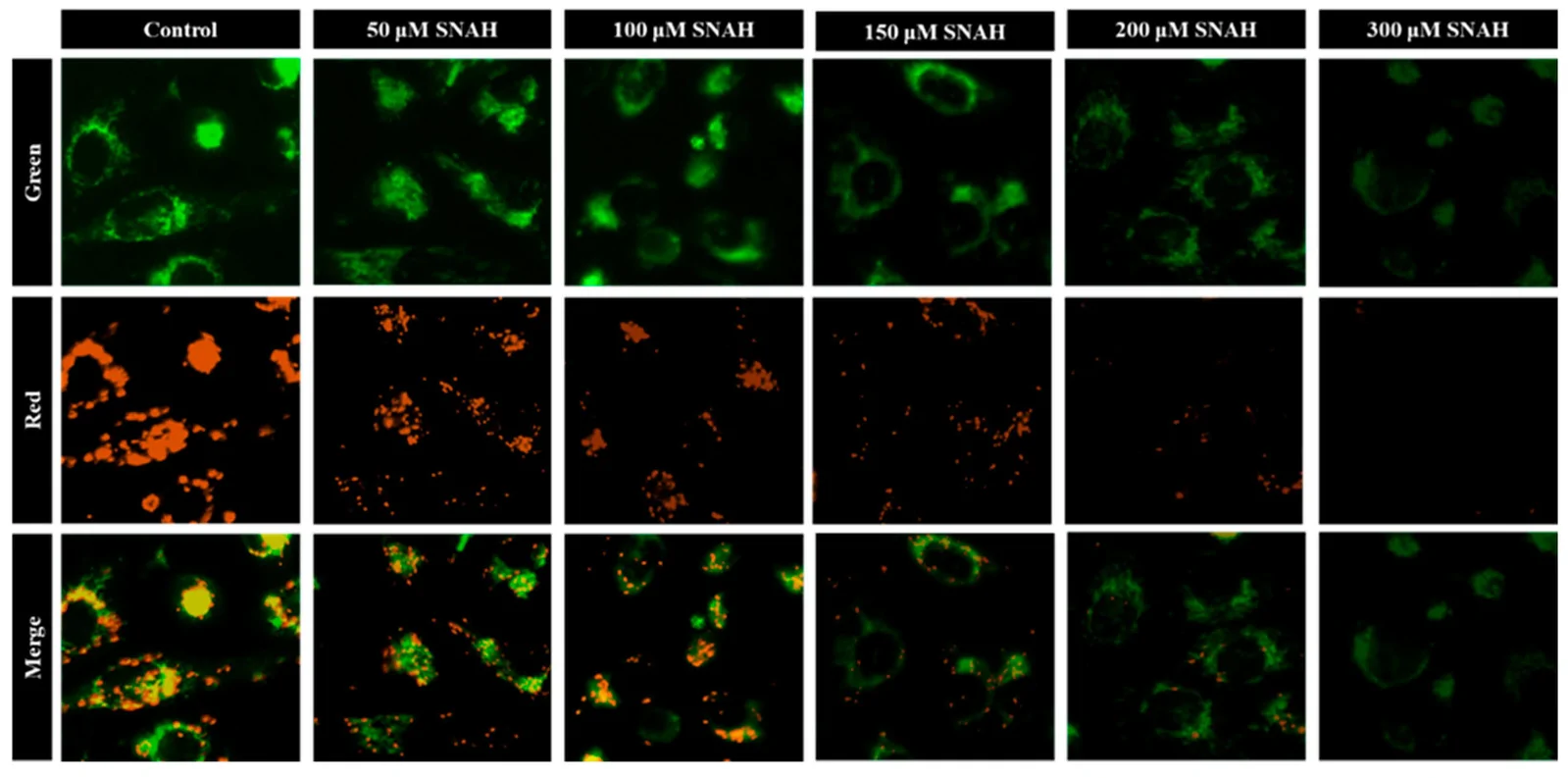
Analysis of mitochondrial membrane potential by JC-1 staining and analysis in MDA-MB-231 cells treated with SNAH. Changes in red and green fluorescence measured by fluorescence microscopy after JC-1 staining. Mitochondrial depolarization is reflected by a decrease in the red-to-green fluorescence ratio.

The effect of sinapaldehyde (SNAH) on mitochondrial membrane potential (MMP) was evaluated using JC-1 staining. Fluorescence microscopy demonstrated a clear reduction in the ratio of red aggregates (indicative of polarized mitochondria) to green monomers (indicative of depolarized mitochondria) in SNAH-treated cells compared to that in the controls (Figure 12).

### 2.12. Identification of Overlapping Targets Between SNAH and Breast Cancer-Related Genes

**Figure 13:**
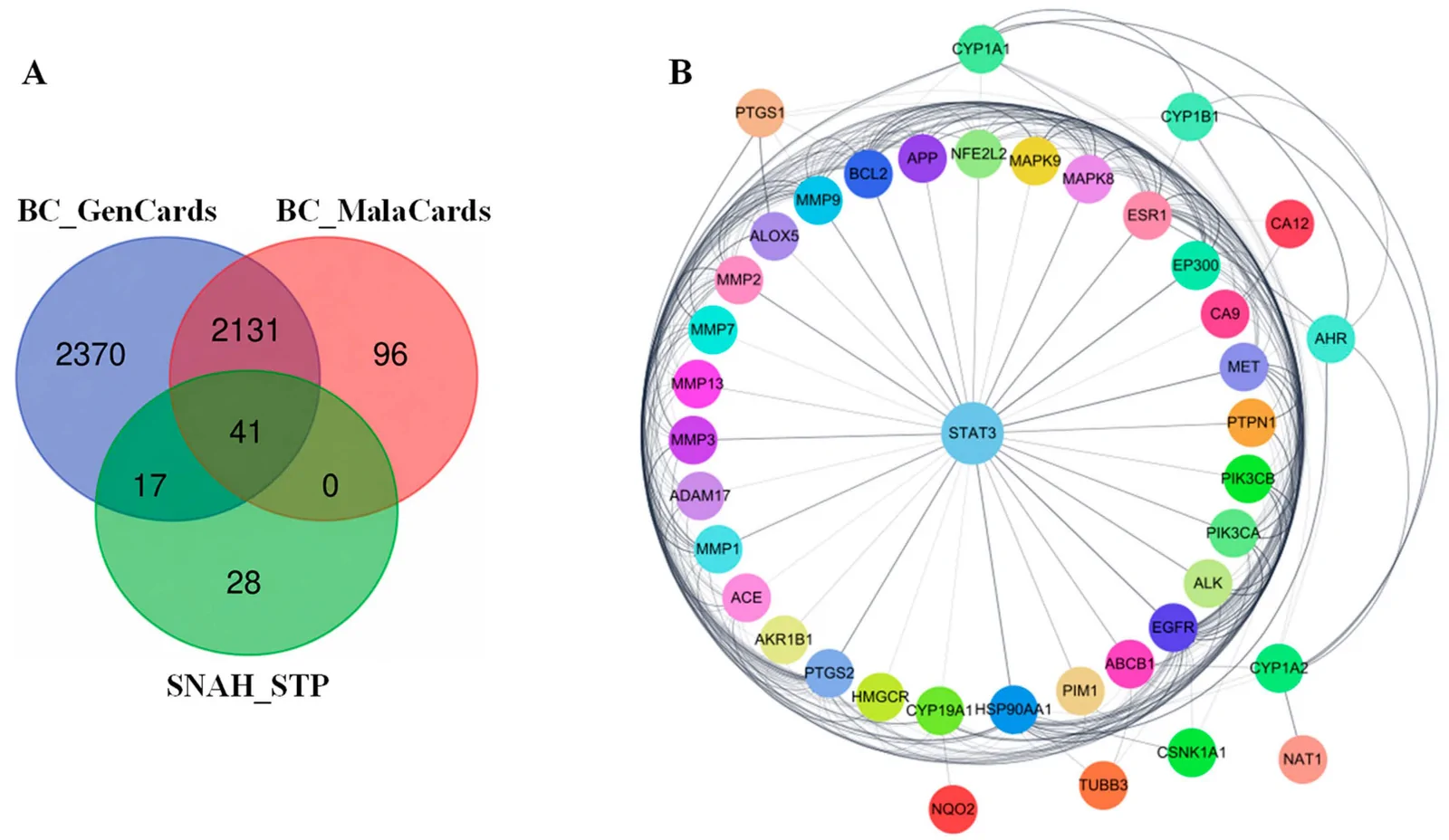
(**A**) Overlapped targets of breast cancer and SNAH targets using a Venn diagram. (**B**) Protein–protein interaction network of intersection targets representing bubbles.

To elucidate the potential molecular mechanisms of SNAH against breast cancer (BC), a comparative target analysis was performed using a Venn diagram. As shown in Figure 13A, a total of 4655 breast cancer-related targets were obtained from the GeneCards (BC_GeneCards) and MalaCards (BC_MalaCards), while 86 targets were associated with SNAH by using the Swisstargetprediction database (SNAH_STP). Intersection analysis revealed 41 common targets shared among the three datasets, representing the core therapeutic targets of SNAH in breast cancer diseases.

### 2.13. Protein–Protein Interaction (PPI) Network

The PPI network was constructed by uploading 41 overlapping targets into the STRING database (https://string-db.org/). Output data was sent to Cytoscape (Ver. 10.1) with the STRING plugin for visualizing the PPI network (Figure 13B). This biological network consisted of interconnected nodes representing functional protein associations. The dense network connectivity reflects the multiple pharmacological actions of SNAH, providing mechanistic insights into its potential therapeutic actions against BC.

### 2.14. Gene Ontology (GO) Enrichment Analysis of SNAH Targets Related to Apoptosis and Breast Cancer

**Figure 14:**
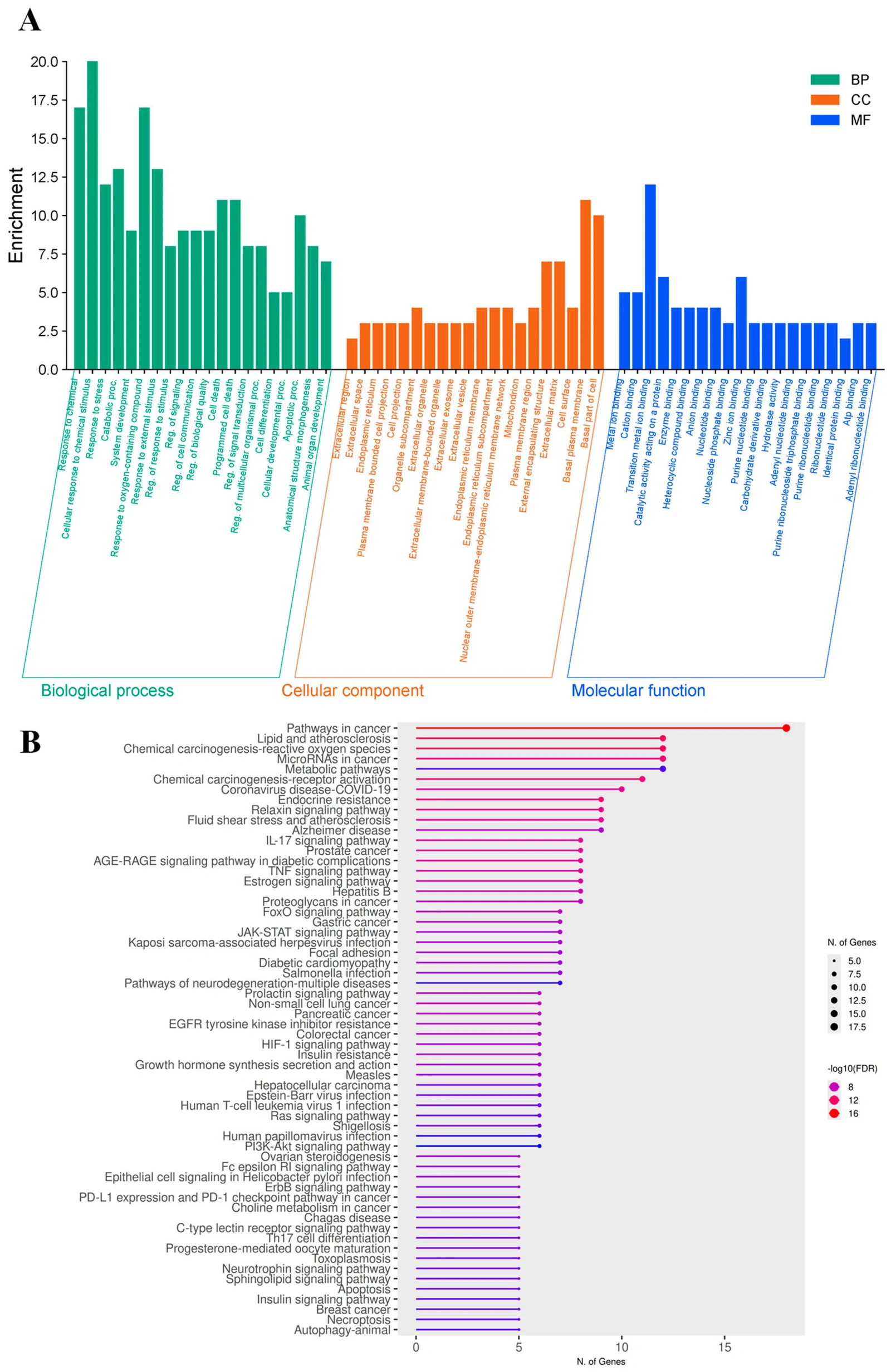
(**A**) GO term analysis (BP: biological function; CC: cellular component; and MF: molecular function). (**B**) KEGG enrichment analysis.

The Gene Ontology (GO) enrichment analysis of the compound–breast cancer target network revealed significant enrichment across biological process (BP), cellular component (CC), and molecular function (MF) categories (Figure 14A). In the BP category, the targets were mainly involved in responses to chemical and external stimuli, oxidative stress, and regulation of signal transduction and programmed cell death. CC enrichment indicated that these targets were predominantly localized in the extracellular region, plasma membrane, endoplasmic reticulum, and mitochondria. In the MF category, enriched terms such as protein binding, transition metal ion binding, and catalytic activity reflected the regulation of key enzymatic and redox-related functions. Collectively, these results suggest that SNAH exerts multi-target effects through modulation of apoptosis, oxidative stress, and signal transduction processes, contributing to the potential therapeutic mechanisms against breast cancer.

### 2.15. KEGG Pathway Enrichment Analysis of SNAH Targets Involved in Apoptosis and Breast Cancer

To further elucidate the molecular mechanisms underlying the anti-breast cancer effects of SNAH, KEGG pathway enrichment analysis revealed that the overlapping targets between SNAH and breast cancer-related genes were significantly associated with multiple cancer-related and inflammation-mediated signaling pathways (Figure 14B). Among the top enriched pathways, “Pathways in cancer,” “Lipid and atherosclerosis,” “Chemical carcinogenesis–reactive oxygen species,” “MicroRNAs in cancer,” and “Metabolic pathways” exhibited the highest enrichment scores and involved the greatest number of genes, suggesting a strong relevance to tumor initiation and progression. Notably, several other pathways such as apoptosis, necroptosis, and autophagy were also significantly enriched, suggesting the involvement of cell death and survival regulation in the therapeutic effects of the compounds. The enrichment of breast cancer, prostate cancer, and colorectal cancer pathways further supports the multi-target and multi-pathway nature of SNAH activity.

### 2.16. Compound–Target–Pathway Network Analysis

**Figure 15:**
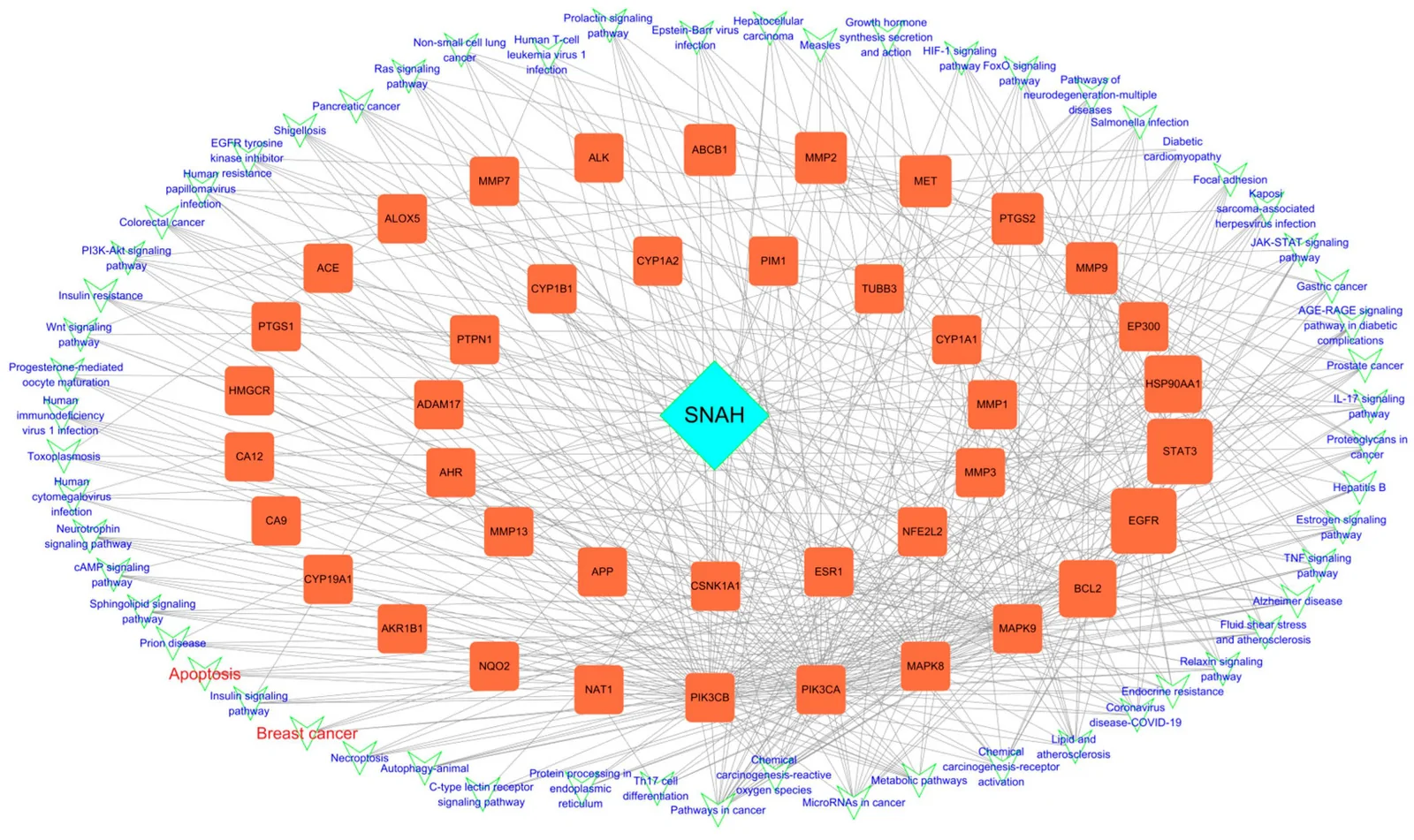
The SNAH–target–pathway network is depicted with green arrows representing pathways and cyan diamonds representing SNAH, respectively. Orange rounded rectangles represent targets, including crucial genes related to bubble sizes.

The compound–target–pathway interaction network revealed that SNAH exhibited extensive multi-target and multi-pathway associations with potential therapeutic relevance to breast cancer (Figure 15). Among these targets, EGFR, STAT3, BCL2, ESR1, and HSP90AA1 were determined to be hub nodes based on central scores with high connectivity, suggesting their central regulatory roles in breast cancer pathophysiology. Given the ER-negative phenotype of MDA-MB-231 cells, the biological relevance of ESR1 should be interpreted cautiously, and other TNBC-associated hubs such as EGFR, STAT3, HSP90AA1, and BCL2 may be more directly involved in the observed anti-cancer effects. These core targets are well-known mediators of cell proliferation and apoptosis inhibition, indicating that SNAH may exert its anti-cancer effects through simultaneous modulation of these critical molecular processes. Therefore, these targets were selected to evaluate their interaction with SNAH through molecular docking.

### 2.17. Molecular Docking Analysis

SNAH was docked into the above key proteins to evaluate its binding affinity through docking scores and interacting with key residues at the binding pocket of proteins (Figure S9, Supplementary Materials). SNAH displayed interactions with EGFR and BLC2 proteins with low binding energies of −8.86, −7.92, and −7.53 kcal/mol, respectively, whereas SNAH docked into STAT3 and HSP90AA1 proteins with docking scores of −6.04 and −5.87 kcal/mol. SNAH formed hydrogen bonds with some residues of the EGFR protein including MET769 and GLU738, and hydrophobic interactions with ALA719, ILE720, LYS721, LEU736, MET742, LEU764, ILE765, THR766, GLN767, LEU820, THR830, ASP831, and PHE832. Moreover, SNAH interacted with amino acids of the BCL2 protein such as TYR108 and PHE112 by forming hydrogen bonds, and with PHE104, SER105, ASP111, MET115, VAL133, VAL134, LEU137, ALA149, PHE150, GLU152, PHE153, and VAL156 by hydrophobic interaction. SNAH showed some interactions with the STAT3 protein by hydrogen bonds with the GLU638 and LYS658 residues and hydrophobic interactions with the VAL637, PRO639, TYR640, GLN644 and TYR657 amino acids. Moreover, SNAH was found to be surrounded by the HSP90AA1 protein with hydrogen bonds formed with the ASN300, VAL331 and PHE337 residues and hydrophobic interactions with the TRP297, SER330, GLU332, GLU336, ARG367, ASP390, SER391 and GLY392 residues.

## 3. Discussion

**Figure 16:**
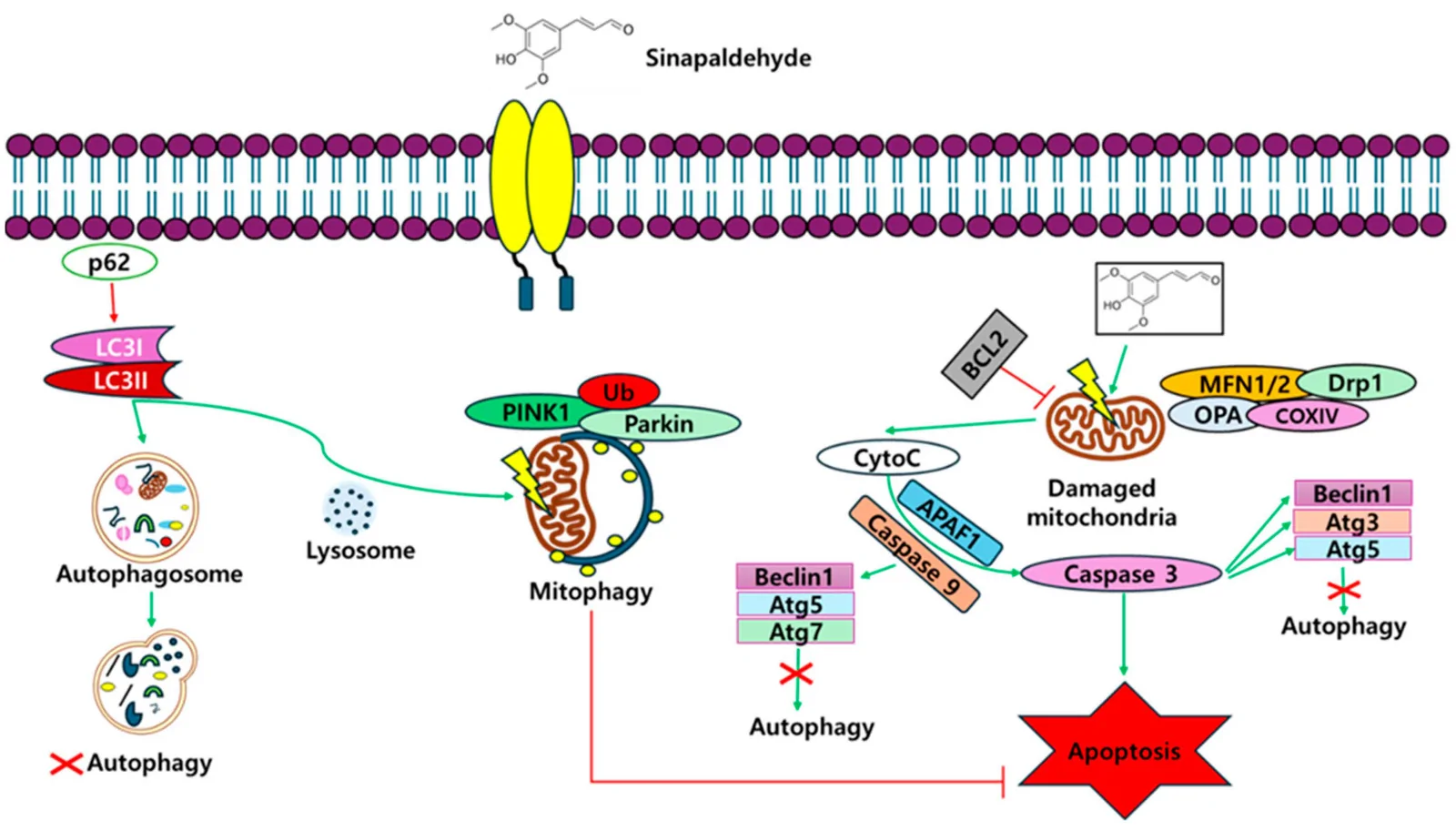
Proposed model of SNAH-induced mitochondrial dysfunction, autophagy/mitophagy-associated responses, and apoptosis in MDA-MB-231 cells. SNAH treatment is accompanied by changes in autophagy-associated markers. Sustained exposure is associated with altered mitochondrial dynamics, including reduced OPA1 expression, and changes in mitophagy-associated signaling. These changes are accompanied by activation of apoptotic pathways and reduced Beclin-1 expression together with altered expression of autophagy-related proteins. SNAH also altered ferritinophagy-associated markers, including FTH1 and NCOA4, as well as PINK1/Parkin-associated responses. Collectively, SNAH-induced mitochondrial dysfunction is associated with alterations in autophagy/mitophagy-related signaling and subsequent activation of apoptosis.

Triple-negative breast cancer (TNBC) is a highly aggressive subtype of breast cancer characterized by the absence of estrogen receptor, progesterone receptor, and HER2 expression and lacks effective targeted therapies. Therefore, novel compounds that can induce cancer cell death via alternative mechanisms are urgently needed [18]. In this study, we investigated the effects of sinapaldehyde (SNAH) in MDA-MB-231 triple-negative breast cancer (TNBC) cells, with particular emphasis on mitochondrial homeostasis, autophagy/mitophagy-associated processes, oxidative stress, and apoptosis. Although the growth-inhibitory activity of sinapaldehyde has previously been reported in several human cancer cell models, the present study extends these observations by examining its effects on mitochondrial function and associated cellular stress and survival pathways in the MDA-MB-231 TNBC model. (Figure 16). These findings provide further insight into the cellular responses associated with SNAH treatment in MDA-MB-231 TNBC cells and provide a basis for further investigation of its potential anti-cancer activity.

Mitochondrial integrity is crucial for cellular homeostasis and mitophagy serves as a key quality-control mechanism for eliminating damaged mitochondria [19]. In response to mitochondrial injury, cells activate mitophagy or undergo mitochondrial fission to remove dysfunctional organelles and sustain energy production [20]. However, when the damage surpasses the cellular capacity for repair, mitochondrial ROS accumulates, pro-apoptotic factors are released, and caspases are activated, culminating in cell death [17]. In our study, NAC attenuated SNAH-induced ROS accumulation, cytotoxicity, caspase cleavage, and mitochondrial morphological alterations, supporting the contribution of oxidative stress to SNAH-induced cellular injury. However, the present data do not establish that mitochondrial dysfunction or autophagy/mitophagy functions as a cytoprotective response. Persistent mitochondrial stress was associated with downregulation of OPA1 and COXIV, indicating altered mitochondrial fusion and inner membrane integrity [21].

Interestingly, SNAH produced differential effects on PINK1 and Parkin at the transcript and protein levels. PINK1 and Parkin transcripts were altered following SNAH treatment, whereas protein-level analysis showed reduced PINK1 and Parkin abundance in the examined fractions. These findings suggest differential regulation of PINK1/Parkin-associated signaling at the transcriptional and post-transcriptional levels. Importantly, neither transcript nor steady-state protein measurements alone are sufficient to determine functional PINK1/Parkin-dependent mitophagic flux. Therefore, the present findings support modulation of PINK1/Parkin-associated responses rather than definitive activation or inhibition of mitophagy.

Caspase activation coincided with reduced Beclin-1 expression, suggesting an association between apoptotic signaling and changes in autophagy-associated processes.

The expression levels of LC3B-I/II and LAMP-1 were significantly increased after 12–48 h of SNAH exposure, indicating increased abundance of autophagy- and lysosome-associated markers. In contrast, Beclin-1 expression progressively decreased over time, coinciding with increased activation of apoptotic markers. This temporal association is consistent with crosstalk between autophagy-associated processes and apoptosis; however, reduced Beclin-1 may also occur secondary to caspase-mediated proteolysis and therefore does not establish a causal transition from protective autophagy to apoptosis (Figure 7).

Collectively, these findings indicate that prolonged SNAH exposure is associated with time-dependent changes in autophagy-associated markers followed by increased activation of apoptotic pathways. These findings are consistent with a temporal association between changes in autophagy-associated markers and subsequent mitochondria-dependent apoptosis in MDA-MB-231 cells; however, a causal transition from autophagy to apoptosis cannot be established from the present data.

Downregulation of Atg3, Atg5, and Atg7 was associated with reduced expression of autophagy-related machinery (Figure S10, Supplementary Materials). These findings align with previous reports that demonstrate autophagy–apoptosis crosstalk, wherein prolonged stress shifts protective autophagy toward a cytotoxic response [17]. Cell death was also associated with increased ROS production and altered iron homeostasis. SNAH-treated cells showed elevated FTH1 expression with relatively unchanged NCOA4 levels, suggesting modulation of ferritinophagy-associated regulation. However, these steady-state changes do not establish impaired ferritinophagic flux or defective ferritin degradation [22] (Figure S11, Supplementary Materials). The contribution of altered ferritinophagy to the observed oxidative and iron-related stress therefore remains to be determined.

Interestingly, while sinapaldehyde altered mitophagy-associated signaling, it simultaneously inhibited colony formation and migration and induced G2/M arrest in MDA-MB-231 cells. Whether altered mitophagic flux contributes directly to SNAH-induced cytotoxicity remains to be determined.

An important limitation of the present study is that the experiments were performed using the MDA-MB-231 TNBC cell model. Furthermore, several mechanistic experiments employed relatively high SNAH concentrations, which should be considered when interpreting the translational relevance of the observed responses. A primary limitation of the current study is that the cellular evaluation was conducted exclusively in MDA-MB-231 cells without comparison to a non-malignant mammary epithelial model (e.g., MCF-10A). Consequently, whether the observed anti-proliferative and apoptotic effects exhibit tumor selectivity remains to be determined. Systematic comparative evaluations against non-malignant counterparts and additional TNBC cell lines will be an essential focus of our follow-up investigations to determine the reproducibility, selectivity, and generalizability of these effects. Therefore, the specificity of the observed effects of SNAH toward TNBC cells, as opposed to a more general cellular response to mitochondrial stress, cannot be established from the present data. Comparative studies using additional TNBC cell lines and non-malignant breast epithelial cells would be necessary to determine the selectivity and generalizability of the observed effects. Nevertheless, the present findings demonstrate that SNAH induces mitochondrial dysfunction and modulates autophagy/mitophagy-associated processes and apoptosis in MDA-MB-231 cells.

Furthermore, the potential involvement of non-canonical pathways in sinapaldehyde-induced mitophagy deserves further attention. Although PINK1/Parkin-independent mitophagy is not well characterized, mechanisms involving alternative lipid sources, such as ER-derived membranes or class II PI3Ks, for PtdIns3P production could compensate for impaired canonical signaling [17,23]. Previous studies have demonstrated that mitophagosome formation can occur via Beclin-1-independent routes, possibly through lipid remodeling or phosphatase activity, altering phosphoinositide pools [24]. Whether sinapaldehyde exploits these alternative mechanisms to initiate early mitophagy remains unknown, and the present data do not establish involvement of these pathways [23].

Network pharmacology revealed SNAH may potentially exhibit anti-breast cancer activity because their predicted targets overlap with a higher number of breast cancer-related targets. The 41 targets refer to a densely connected biological network, with key regulators (EGFR, STAT3, BCL2, ESR1, and HSP90AA1) as central nodes. This observation suggests that SNAH may modulate breast cancer points rather than acting against a single protein. MDA-MB-231 cells belong to the triple-negative breast cancer subtype and exhibit minimal or absent ERα expression. Therefore, the appearance of ESR1 as a highly connected node likely reflects its established role in breast cancer biology at the disease-network level rather than as a direct functional driver in this TNBC model. In contrast, EGFR, STAT3, HSP90AA1, and BCL2 have been extensively implicated in TNBC progression, survival signaling, apoptosis resistance, and mitochondrial homeostasis. Notably, the experimentally observed suppression of BCL2 expression, activation of caspase-dependent apoptosis, mitochondrial dysfunction, and disruption of survival mechanisms are more consistent with these TNBC-associated pathways than with classical estrogen receptor signaling. Molecular docking analysis indicated that SNAH strongly bound into (EGFR, STAT3, BCL2, and HSP90AA1) proteins (docking scores less than −5.87 kcal/mol) by interacting with key amino acids surrounding the binding poses of proteins.

Collectively, our findings highlight the multifaceted roles of mitophagy and autophagy in SNAH-treated TNBC cells. Collectively, our findings indicate that SNAH-induced mitochondrial dysfunction is accompanied by alterations in PINK1/Parkin-associated signaling and autophagy-associated markers, together with subsequent activation of intrinsic apoptosis. However, the present data do not establish whether altered PINK1/Parkin-dependent mitophagic flux or autophagic flux causally contributes to SNAH-induced apoptosis [25]. Further investigation of the interplay between ROS signaling, iron metabolism, and non-canonical mitophagy pathways could yield novel strategies for enhancing the efficacy of sinapaldehyde or similar agents in drug-resistant cancer.

## 4. Materials and Methods

### 4.1. Identification and Separation of SNAH from PS Extract

The dried pistachio shells (1 kg) were extracted with 10 L of ethanol. The extract solution was dried by using a rotary evaporator. The analytical methods to identify SNAH from PS extract were conducted by following our previous method without modification [14]. Then, the total extract was suspended in water and partitioned with EtOAc (E) to get the E fraction. This fraction was solubilized with 100% methanol before loading into a silica gel column (25 × 350 mm) and eluted with a chloroform–methanol solution (5:1) to obtain ten fractions (E1–E10). Fraction E2 was subjected to an ODS C_18_ column (5 × 7 cm) and an eluting gradient ratio of acetonitrile (30%, 50%, 70%, and 100%) in water to get four sub-fractions (A–D) and further purified by using the same experimental conditions on an Agilent HPLC instrument (1260 Infinity II Prep-HPLC System, Agilent, Santa Clara, CA, USA, in Bio-Health Materials Core-Facility, Jeju National University). Chromatographic separation was conducted on an Agilent ODS C_18_ column (10 × 250 mm, Agilent, Santa Clara, CA, USA) using a gradient solvent system of methanol in water from 10% to 100% for 30 min with compound RT10.8 (*t*_R_ 10.8 min). The injection volume was 500 μL; the flow was 5 mL/min. UV detection was set at 215, 254, and 320 nm.

### 4.2. Reagents and Antibodies

Bafilomycin (Baf-1) was purchased from MedChemExpress (Princeton, NJ, USA). The WST assay was purchased from DoGenBio (Seoul, Republic of Korea). Mitochondrial division inhibitor (mdivi-1), N-acetylcysteine (NAC), DMSO, Triton X-100, and paraformaldehyde (PFA) were obtained from Sigma-Aldrich (St. Louis, MO, USA). Hoechst 33342, JC-1, FXCycle PI/RNase staining solution cell cycle assay kit, CellRox Green, and BCA protein assay kits were from Thermo Fisher Scientific (Waltham, MA, USA). The APC/Annexin V Apoptosis Detection Kit with PI was obtained from BioLegend (San Diego, CA, USA). MitoView Green was purchased from Biotium (Fremont, CA, USA). The Xpert Duo Inhibitor Cocktail Solution (100X) was obtained from GenDEPOT (Katy, TX, USA). FeRhoNox-1 was obtained from GORYO Chemical (Hokkaido, Japan) and MitoTracker Red CMXRos was acquired from Cell Signaling Technology (Beverly, MA, USA). TOPscript RT DryMIX (dT18/dN6), TOPreal SYBR Green qPCR PreMIX, and EZ Total RNA Miniprep kits were obtained from Enzynomics (Daejeon, Republic of Korea). Antibodies against Caspase-3 (#9662), Caspase-9 (#9502), cleaved caspase-3 (#9664), cleaved caspase-9 (#7237), Bax (#2774), Bcl-2 (D55G8) (#4223), p-Parkin (ser65) (#36866), SQSTM1/p62 (#5114), OPA (D6U6N) (#80471), COXIV (3E11) (#4850), Drp1 (D6C7) (#8570), NCOA4 (E8H8Z) (#66849), FTH1 (D1D4) (#4393), Atg7 (D12B11) (#8558), Atg5 (D5F5U) (#12994), Atg3 (#3415), Beclin (D40C5) (#3495), and LC3A/B (D3U4C) (#12741) were obtained from Cell Signaling Technology (Beverly, MA, USA). β-actin, Pink1 (C-3) (#518052), Parkin (PRK8) (#32282), and LAMP-1 (H4A3) (#20011) antibodies were from Santacruz Biotechnology (Dallas, TX, USA). IgG antibodies against rabbits and mice were purchased from LICORbio (Lincoln, NE, USA) (Table S2, Supplementary Materials).

### 4.3. Cell Culture

The MDA-MB-231 human breast cancer cell line was obtained from the Korea Cell Line Bank (Seoul, Republic of Korea). Cell lines were cultured in RPMI (Corning, NY, USA) supplemented with 10% (*v*/*v*) fetal bovine serum (GenDEPOT, Katy, TX, USA) and 1% (*v*/*v*) penicillin–streptomycin (Gibco, Thermo Fisher Scientific) and kept at 37 °C in a humidified incubator with 5% CO_2_.

### 4.4. Cell Viability Assay

Cells (1.5  ×  10^6^/plate) were seeded into 96-well plates and cultured overnight. After SNAH treatment for 24 h, the drug-containing medium was removed, and the cells were washed using 1× PBS. Then, 100 µL of WST (10%) was added to each well and incubated at 37 °C for 1 h; absorbance was then measured at 450 nm using a microplate reader (VersaMax ELISA Microplate; Molecular Devices, San Jose, CA, USA). Each data series was obtained in triplicate.

### 4.5. Antioxidant Assay

Antioxidant experiments were performed using 2,2-diphenyl-1-picrylhydrazyl (DPPH), 2,2′-azino-bis (3-ethylbenzothiazoline-6-sulfonic acid) (ABTS), and ferric reducing antioxidant power (FRAP) assays. In a 96-well plate, different concentrations of SNAH and antioxidants (ascorbic acid (ASA) and butylated hydroxyanisole (BHA)) were incubated for 30 min with 200 µM DPPH solution, for 5 min with 200 µM ABTS solution, and for 30 min with 200 µM FRAP solution. A VersaMax plate reader (Molecular Devices, San Jose, CA, USA) was used to measure the absorbance at OD_517_, OD_734_, and OD_593_ for the DPPH, ABTS, and FRAP assays, respectively. DPPH scavenging activity was determined using the following equation: DPPH scavenging activity (%) = {1 − [(Sample-Blank)/Control]} multiplied by 100. ABTS scavenging activity (%) = {1 − [(Sample-Blank)/Control]} × 100.

### 4.6. Colony Formation Assay

MDA-MB-231 cells were seeded at a low density (3  ×  10^4^/plate) in a six-well plate and treated with varying doses of sinapaldehyde in RPMI medium. After 24 h, the medium was replaced with fresh medium, and the cells were allowed to grow for five days. The colonies were then stained, counted, and evaluated using the NICE program (version 1.1.0).

### 4.7. Cell Migration Assay

MDA-MB-231 TNBC cells were seeded in a six-well plate (5  ×  10^6^/plate) and cultured to 90% confluence. A scratch was produced throughout the cell layer using a sterile 10 µL micropipette tip. After washing with 1× PBS, the cells were treated with SNAH. The wounded areas were photographed under a light microscope at 10× magnification after 6, 12, 15, and 20 h.

### 4.8. Cell Cycle Assay

Following SNAH treatment for the appropriate duration, cells were harvested and stained using the FxCycle PI/RNase staining solution cell cycle assay kit, according to the manufacturer’s protocol. A CytoFLEX flow cytometer (Beckman Coulter, Inc., Brea, CA, USA) was used to investigate various phases of the cell cycle. The results were quantitatively evaluated using CytExpert 1.0 (Beckman Coulter, Inc., CA, USA).

### 4.9. Apoptosis Detection by Flow Cytometry

Following the prescribed SNAH treatment duration, the cells were harvested and stained using an APC Annexin V-APC/PI staining assay kit according to the manufacturer’s recommendations. The apoptotic rates were analyzed using a CytoFLEX flow cytometer (Beckman Coulter, Inc., CA, USA). The results were quantitatively evaluated using CytExpert 1.0 (Beckman Coulter, Inc., CA, USA).

### 4.10. Real-Time RT-qPCR

Total RNA was extracted using the EZ Total RNA Miniprep kit and cDNA was synthesized using TOPscript RT DryMIX (dT18/dN6). RT-qPCR was then performed using the TOPreal SYBR Green qPCR kit (Enzynomics, Republic of Korea). The list of primer sequences used is provided in Supplementary Table S1. The housekeeping gene β-actin served as an internal control for RT-qPCR.

### 4.11. Cell Staining Assay

Cells (1.5 × 10^6^ cells/plate) were grown in 96-well plates and cultured overnight. Following the indicated procedures, cells were stained with appropriate reagents for various experimental purposes. To detect mitochondrial morphology, cells were treated with MitoTracker^®^ Red CMXRos (100 nM) for 15 min at 37 °C in the dark followed by MitoViewTM Green (50 nM) for 1 h at 37 °C. To observe the morphological changes associated with apoptosis, Hoechst 33342 (1 μg/mL) staining was performed for 15 min at room temperature in the dark. FeRhoNox-1 (5 μM) staining for 1 h at 37 °C in the dark was used to detect labile Fe2+ ions by orange fluorescence. CellROX Green staining for 30 min at 37 °C in the dark was performed to detect cellular ROS buildup. Finally, cellular fluorescence was examined using a phase-contrast fluorescence microscope (CYTATION5, Agilent Technologies Inc, Santa Clara, CA, USA, in Bio-Health Materials Core-Facility, Jeju National University).

### 4.12. Immunofluorescence Assay

The cells were cultured in 96-well plates and treated with SNAH. The cells were then fixed with 4% PFA and treated with 0.1% Triton X-100. The cells were incubated with NH_4_Cl and blocked with 3% BSA. The cells were then incubated overnight at 4 °C with a primary antibody against LC3 and LAMP, and then with a fluorescent secondary antibody for 1 h at room temperature before labelling with DAPI for 15 min. Finally, cellular fluorescence was assessed using a phase-contrast fluorescence microscope (CYTATION5, Agilent Technologies Inc, Santa Clara, CA, USA, in Bio-Health Materials Core-Facility, Jeju National University).

### 4.13. Western Blotting

Cells were trypsinized and lysed in RIPA lysis buffer (Xpert Duo Inhibitor Cocktail Solution) to extract total cellular proteins. MDA-MB-231 cells were resuspended in cytosolic extraction buffer (10 mM HEPES, 1.5 mM MgCl2, 10 mM KCL, 0.05% Nonidet P-40, and DW). The cytosolic protein fraction was isolated by collecting the supernatant after centrifugation. To extract nuclear proteins, the cell pellets were lysed using RIPA lysis buffer (containing Xpert Duo Inhibitor Cocktail Solution). Protein concentrations were determined using the bicinchoninic acid assay. Cellular proteins were separated using SDS-PAGE and transferred to PVDF membranes (Millipore, Burlington, MA, USA). The membranes were incubated overnight at 4 °C with the primary antibodies. After washing, the blots were probed with IRDye 680RD and 800 W secondary antibodies and the resulting signals were quantified using ODYSSEY CLx (LI-COR, Lincoln, NE, USA).

### 4.14. Data Acquisition of Compound and Disease-Related Targets

Sinapaldehyde was inserted into the PubChem web server (https://pubchem.ncbi.nlm.nih.gov/, accessed on 12 July 2026) to obtain the SMILES before importing it into the Swisstargetprediction database (https://www.swisstargetprediction.ch/predict.php, accessed on 13 July 2026) to collect the compound targets. Breast cancer-related targets were defined by using the GeneCards and MalaCards databases through entering “Breast cancer” as a key word with species set as “*Homo sapiens*”. The overlapping targets were identified by using the Venn diagram for further analysis [26].

### 4.15. Protein–Protein Interaction (PPI) Network Construction

The overlapping targets were imported into the STRING database by setting a high confidence level of >0.7 before exporting output data to the STRING plugin Cytoscape software for visualization of biologically interacting networks of these targets. Central nodes were determined by applying the Cytohubba plugin using the degree algorithm [27].

### 4.16. Gene Ontology (GO) and Kyoto Encyclopedia of Genes and Genomes (KEGG) Pathways

The enrichment analysis for GO and KEGG targets was performed to elucidate the mechanisms of actions underlying the anti-breast cancer property of SNAH, utilizing the ShinyGO (version 0.82) online tool (http://bioinformatics.sdstate.edu/go/, accessed on 15 July 2026). The gene symbols associated with prevalent SNAH targets were identified by applying the analysis to ‘*Homo sapiens*’ with a stringent FDR threshold of 0.05 for the enrichment assessment of biological processes (BP), cellular components (CC), molecular functions (MF), and KEGG pathways. The enrichment outcomes were subsequently integrated into the bioinformatics platform (https://www.bioinformatics.com.cn/en, accessed on 17 July 2026) to facilitate the creation of visualization maps [28].

### 4.17. Construction and Analysis of a Compound–Target–Pathway (CTP) Network

To gain an integrated view, a CTP network was established by combining data on the PPI network, the target genes, and the enrichment of KEGG signaling pathways. Cluster analysis and visualization were performed by using the Cytoscape software [29].

### 4.18. Molecular Docking

The SNAH configuration structure was assembled by using the PubChem database before transferring its sdf format into pdbqt format by using the Openbabel program. The proteins are BCL2 (PDB: 6O0K) [30], EGFR (PDB: 1M17) [31], STAT3 (PDB: 6NJS) [32], and HSP90AA1 (PDB: 3Q6N) [33], obtained from the protein data bank (https://www.rcsb.org/). The ligand and proteins were prepared by using MGL tools 1.5.6. Binding energies were calculated by using Autodock 4.2. Visualization of interactions between the ligand and each protein was performed using the Discovery studio software version 2021 [34].

### 4.19. Statistical Analysis

The data are presented as mean ± SD. Statistical analyses were performed using one-way analysis of variance (ANOVA), followed by Tukey’s multiple-comparisons test, where appropriate. When comparisons were made against a single control group, Dunnett’s multiple-comparisons test was used where indicated. For experiments involving two independent factors, two-way ANOVA followed by Tukey’s multiple-comparisons test was used. The wound-healing assay was analyzed using two-way repeated-measures ANOVA followed by a multiple-comparisons test. *p*-values less than 0.05 were considered statistically significant. All statistical analyses were performed using GraphPad Prism 8.0.2 software.

## 5. Conclusions

Sinapaldehyde induces mitochondrial dysfunction and apoptosis in MDA-MB-231 TNBC cells and is associated with substantial alterations in autophagy- and mitophagy-related cellular responses. SNAH treatment resulted in mitochondrial depolarization, increased ROS production, altered mitochondrial dynamics, and activation of the intrinsic apoptotic pathway, as evidenced by increased Annexin V-positive cells and caspase-9 and caspase-3 cleavage. SNAH also altered the expression of PINK1, Parkin, LC3B, Beclin-1, LAMP1, ATG proteins, FTH1, and NCOA4, indicating modulation of mitochondrial quality-control and autophagy-associated processes. Pharmacological modulation of mitochondrial fission and lysosomal degradation further demonstrated an interaction between these cellular processes and SNAH-induced cytotoxicity. However, the present data do not establish functional inhibition of PINK1/Parkin-dependent mitophagic flux, a definitive transition from protective autophagy to apoptosis, or impaired ferritinophagic flux. These mechanisms require further validation using direct flux assays, pathway-specific approaches, and additional cellular models. Furthermore, because the present study was performed using a single TNBC cell line and several mechanistic experiments employed relatively high SNAH concentrations, additional TNBC and non-malignant breast epithelial models will be required to determine the selectivity, reproducibility, and generalizability of the observed effects.

## Data Availability

The original contributions presented in this study are included in the article/Supplementary Material. Further inquiries can be directed to the corresponding author.

## Supplementary Materials

The following supporting information can be downloaded at https://www.mdpi.com/article/10.3390/ijms27209359/s1.
