# Supplementary material for: Sinapaldehyde Induces Mitochondrial Dysfunction and Modulates Autophagy/Mitophagy-Associated Signaling and Apoptosis in MDA-MB-231 Triple-Negative Breast Cancer Cells
Source: Int J Mol Sci. 2026 Oct 21;27(20):9359. doi: 10.3390/ijms27209359 (PMC13704905; doi:10.3390/ijms27209359)
Supplement: Supplementary file 1 [file ijms-27-09359-s001.zip › ijms-4553727-supplementary.pdf]

Supplementary materials

High mass spectrometry

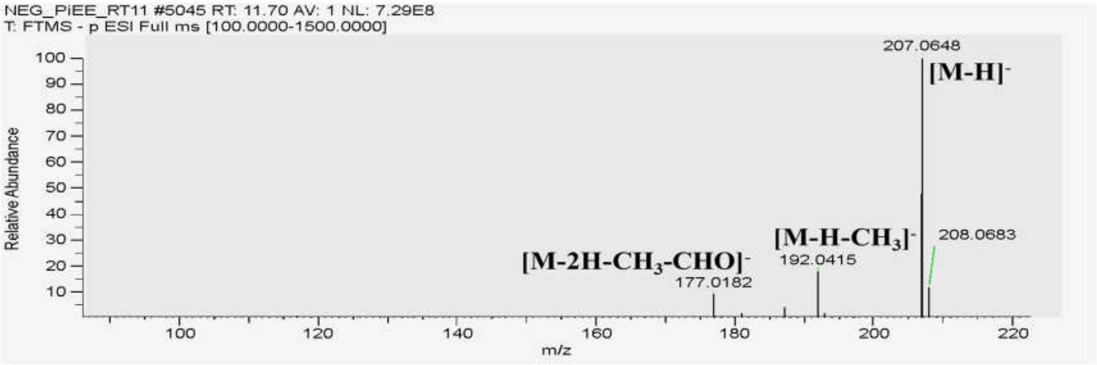

Figure S1. HR-ESI-MS/MS spectrum and MS fragmentation pathway of RT10.8.

NMR spectroscopic data

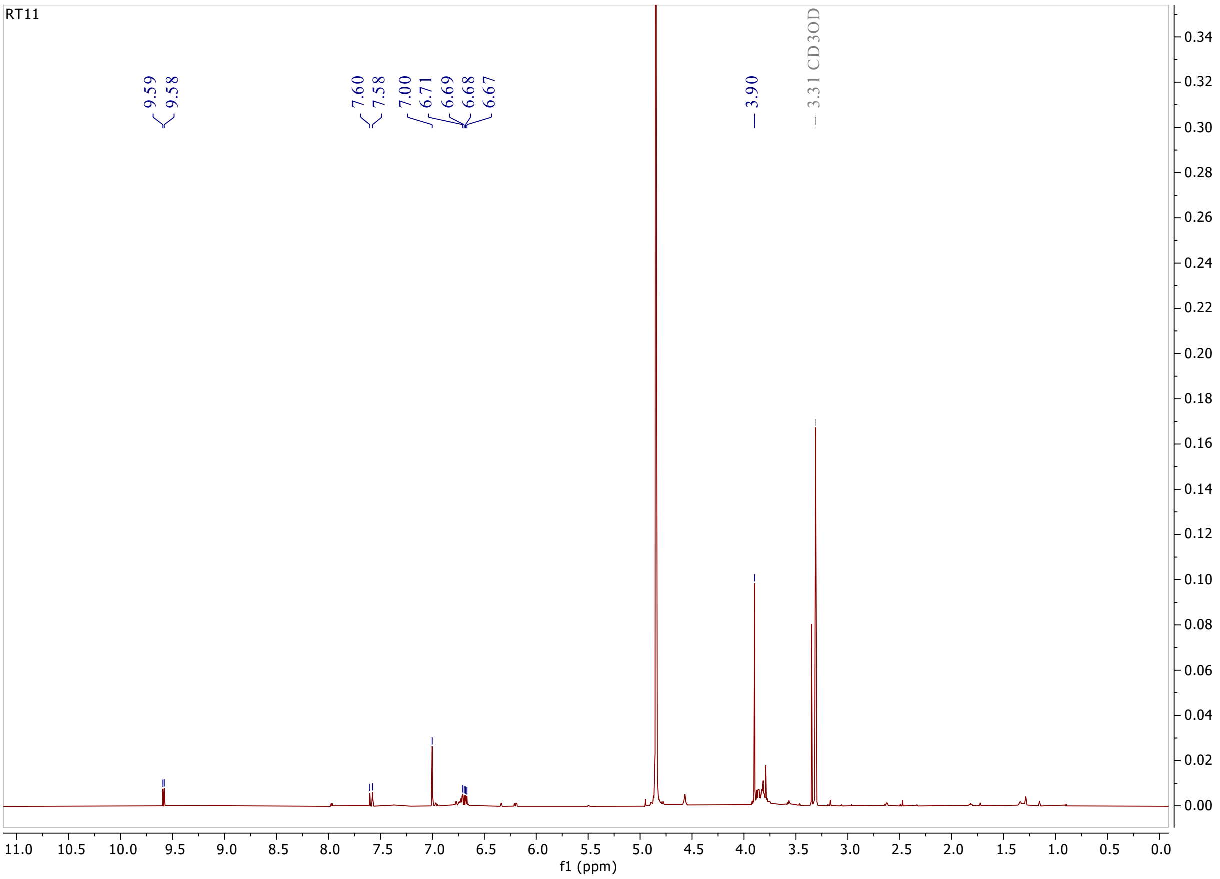

Figure S2. ^{1}H-NMR spectrum of RT10.8 measured in CD_3OD at 600 Mhz.

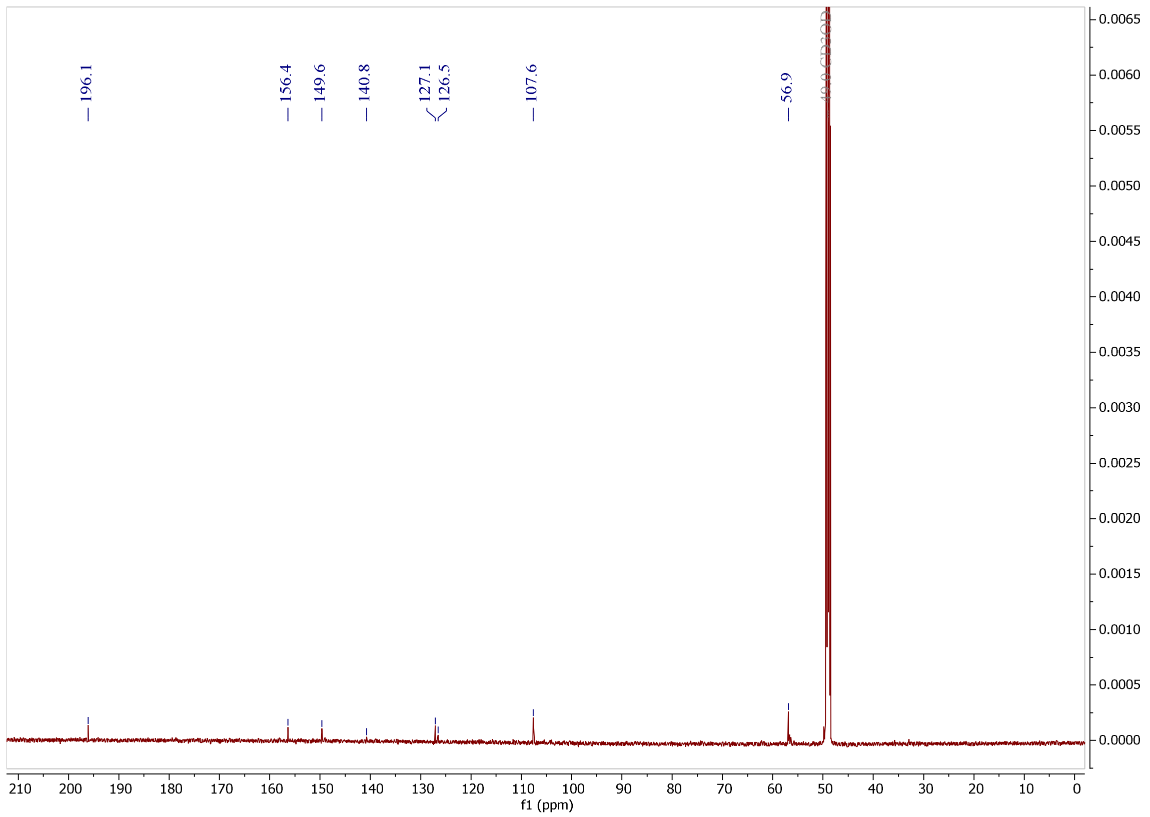

Figure S3. ^{13}C-NMR spectrum of RT10.8 measured in CD_3OD at 150 Mhz.

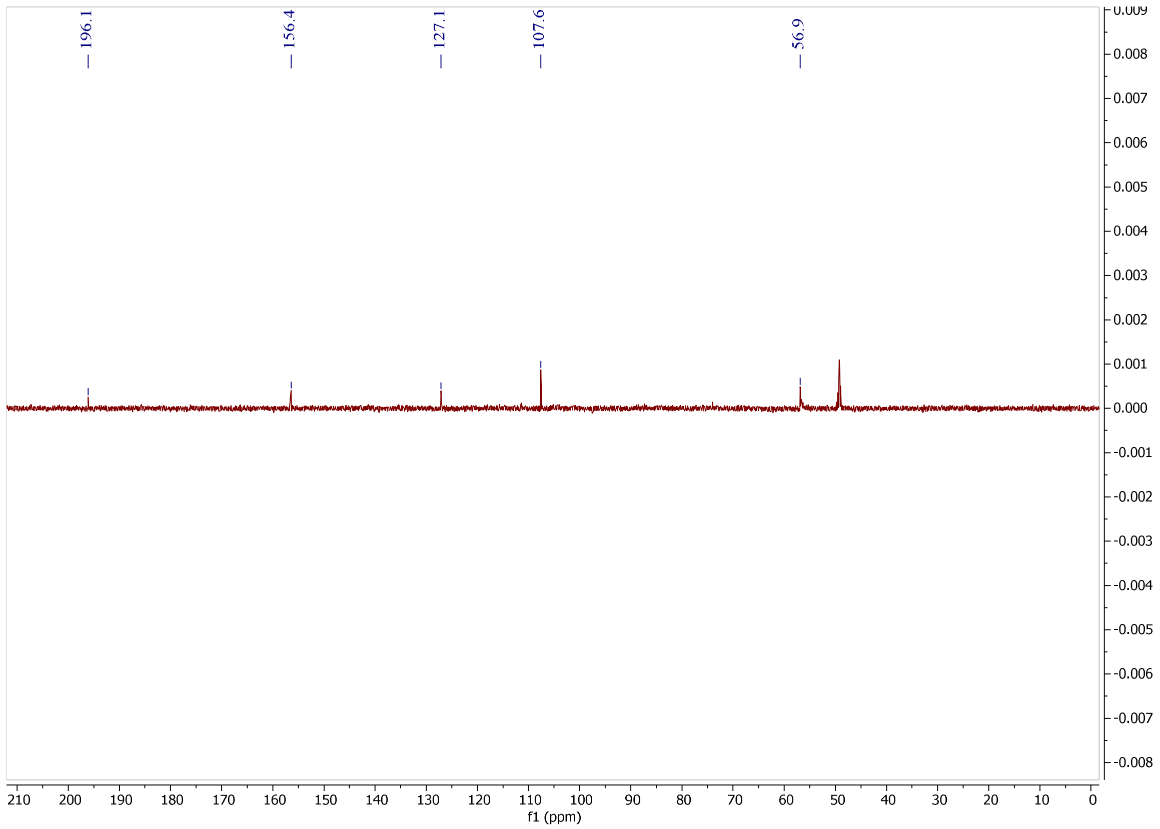

Figure S4. Dept spectrum of RT10.8 measured in CD_3OD at 150 Mhz.

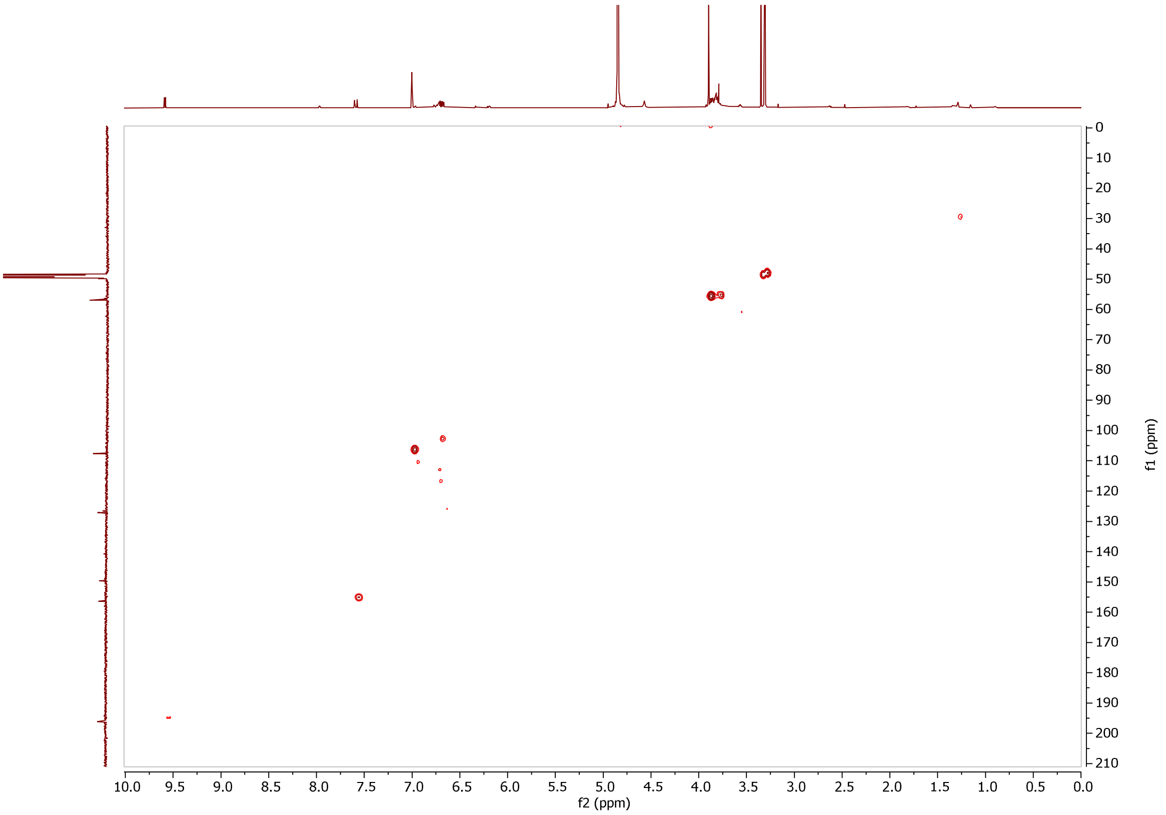

Figure S5. **^{1}H–^{13}C HMQC spectrum of RT10.8 measured in CD_3OD.**

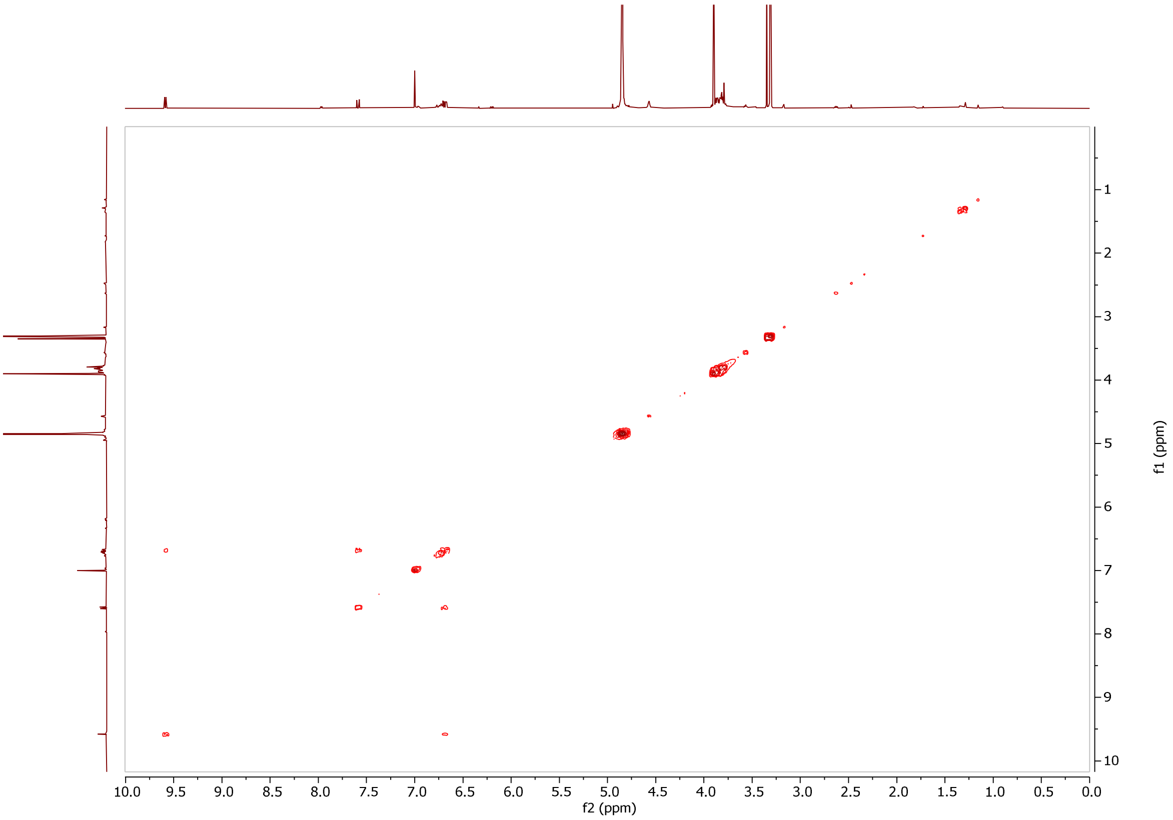

Figure S6. **^{1}H–^{1}H HMQC spectrum of RT10.8 measured in CD_3OD.**

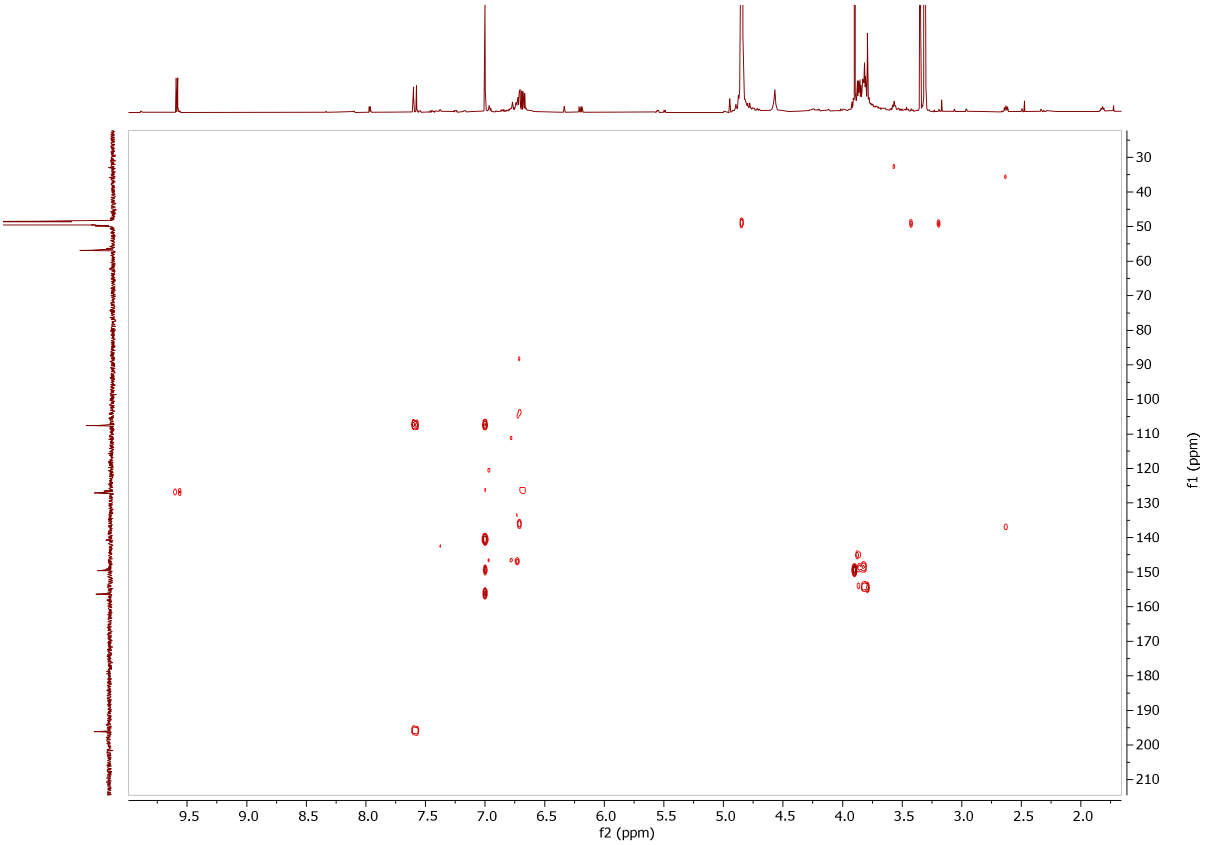

Figure S7. ^1H–^{13}C **HMBC spectrum of RT10.8 measured in** CD_3OD.

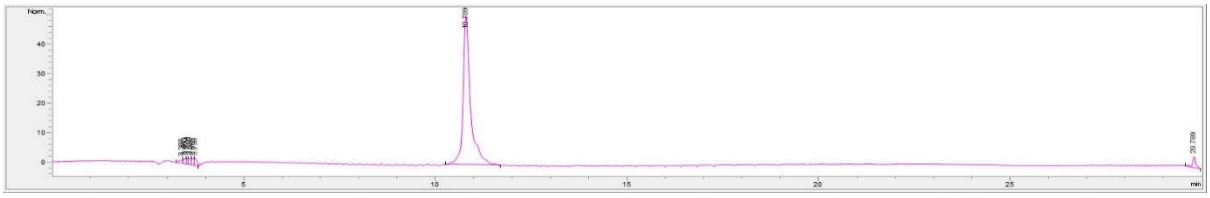

Figure S8. Purity of isolated compound (RT10.8) was 98.6%. HPLC analysis was performed using an Agilent HPLC instrument (1260 InfinityII Prep-HPLC System, Agilent, California, USA in Bio-Health Materials Core-Facility, Jeju National University). Chromatographic separation was conducted using an Aglient ODS C_{18} column (4.6 × 250 mm, particle size 5 μm). The sample was prepared and filtered through a 0.2-μm syringe filter prior to HPLC analysis. The injection volume for HPLC isolation was 10 μL, the flow was 1 mL/min, the column was at room temperature, and the detection was performed at 254 nm. The mobile phase was composed of water (solvent A) and methanol (solvent B). For gradient elution, solvent B was initially set at 10%, increased to 100% at 30 min.

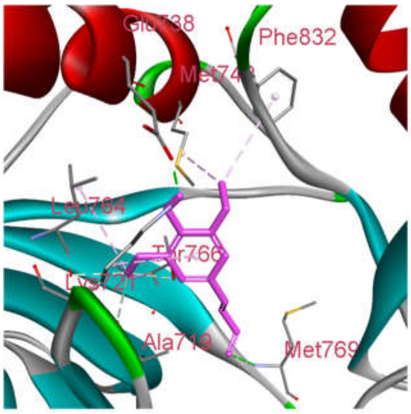

A

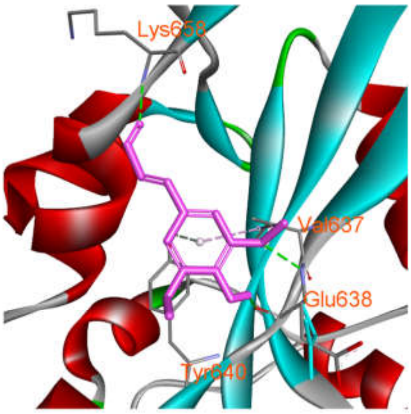

B

Table. Binding energies of SNAH docked into target proteins

Protein	Scores (kcal/mol)
EGFR	-8.86
STAT3	-6.04
BCL2	-7.92
HSP90AA1	-5.87

C

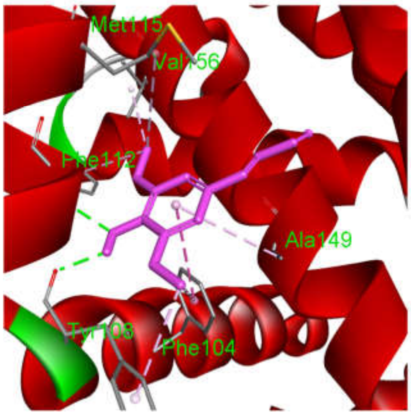

D

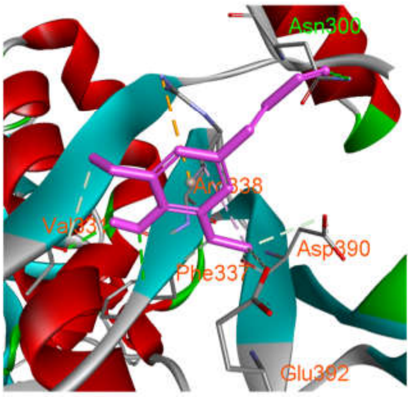

Figure S9. Visualization of SNAH interacting with target proteins (A: EGFR; B: STAT3; C: BCL2; and D: HSP90AA1).

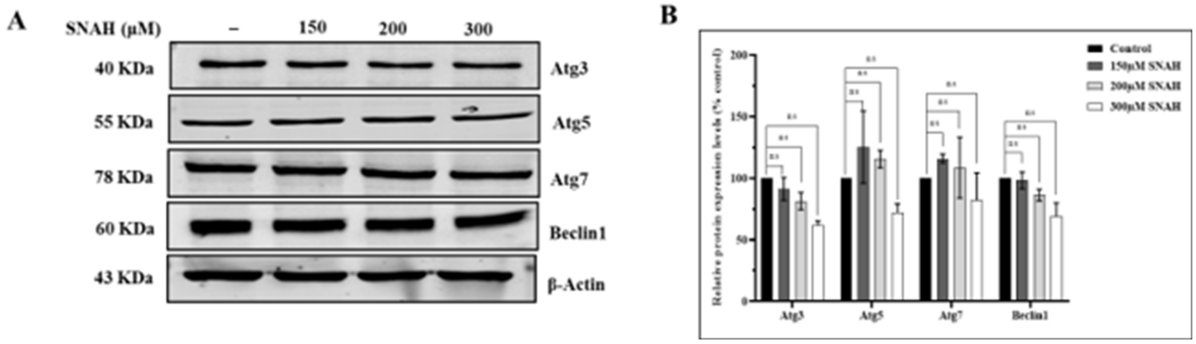

Figure S10. Sinapaldehyde (SNAH) induces mitochondrial dysfunction in MDA-MB-231 cells through a non-classical autophagy pathway. (A) Western blot analysis of autophagy-related protein expression levels in cells treated with 150 µM and 300 µM SNAH. β-actin was used as a loading control. (B) Densitometric analysis of protein bands, normalized to β-actin, showing relative protein expression levels compared to the control.

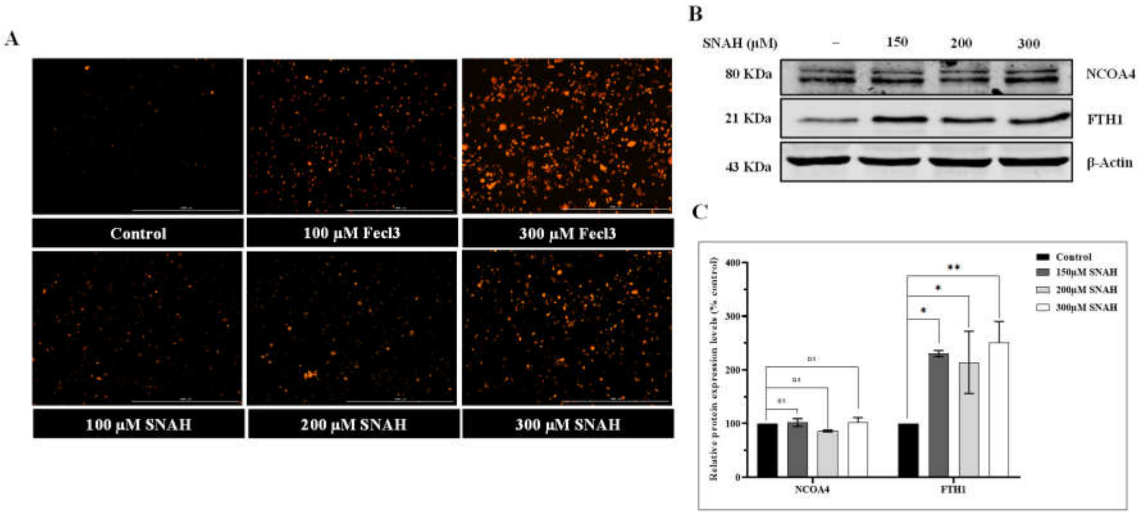

Figure S11. Sinapaldehyde (SNAH) alters ferritinophagy-associated markers in MDA-MB-231 cells. (A) FeRhoNOX-1 staining to check labile Fe^{2+} levels. (B) Western blot analysis of ferritinophagy-associated proteins NCOA4 and FTH1 in SNAH-treated cells. β-actin was used as the loading control. (C) Densitometric analysis of protein bands, normalized to β-actin.

Table S1 – Specific primer sequences used for Real-time RT-qPCR

Genes		Primers
Bak	F: 5′-3′	GATCCCGGCAGGCTGATCCC
	R: 5′-3′	TCCTGTTCCTGCTGATGGCGG
Bad	F: 5′-3′	CGAGATCGGGCTTGGGGTGAG
	R: 5′-3′	CTGGGCCCTCATCTGTCTGCC
Bcl-xl	F: 5′-3′	GGAGAACGGCGGCTGGGATA
	R: 5′-3′	GGCCACAGTCATGCCCGTCA
Mcl-1	F: 5′-3′	AGGGCGACTTTTGGCCACCG
	R: 5′-3′	TGCCTTGGAAGGCCGTCTCG
PINK1	F: 5′-3′	CGTTGGACACGAGACGCTTGCA
	R: 5′-3′	GCAATGTAGGCATGGTGGCTTCATAC
Parkin	F: 5′-3′	ATCGACCTCCACTGGGAAG
	R: 5′-3′	GCGTAGGTCCTTCTCGACC
VDAC1	F: 5′-3′	CAACACGGAGACCACCAAAG
	R: 5′-3′	CACAGCCCAGGTTGATATGC
LC3B2	F: 5′-3′	ACCATGCCGTCGGAGAAGAC
	R: 5′-3′	TCTCGAATAAGTCGGACATCTTCTACTCT
Tom20	F: 5′-3′	CCACCTGACAAATGCAATCG
	R: 5′-3′	TTCCACATCATCCTCAGCCA
FIS1	F: 5′-3′	CCTGGTGCGGAGCAAGTACAA
	R: 5′-3′	TCCTTGCTCCCTTTGGGCAG

Table S2 – List of reagents, assay kit, and antibodies.

Antibody/Reagent/ Asasay kit	Catalog Number	Manufacturer (City, State, USA)
Caspase-3	#9662	Cell Signaling Technology (Beverly, MA, USA)
Caspase-9	#9502	Cell Signaling Technology (Beverly, MA, USA)
Cleaved caspase-3	#9664	Cell Signaling Technology (Beverly, MA, USA)
Cleaved caspase-9	#7237	Cell Signaling Technology (Beverly, MA, USA)
Bax	#2774	Cell Signaling Technology (Beverly, MA, USA)
Bcl-2 (D55G8)	#4223	Cell Signaling Technology (Beverly, MA, USA)
p-Parkin (Ser65)	#36866	Cell Signaling Technology (Beverly, MA, USA)
SQSTM1/p62	#5114	Cell Signaling Technology (Beverly, MA, USA)
OPA1 (D6U6N)	#80471	Cell Signaling Technology (Beverly, MA, USA)
COXIV (3E11)	#4850	Cell Signaling Technology (Beverly, MA, USA)
Drp1 (D6C7)	#8570	Cell Signaling Technology (Beverly, MA, USA)
NCOA4 (E8H8Z)	#66849	Cell Signaling Technology (Beverly, MA, USA)
FTH1 (D1D4)	#4393	Cell Signaling Technology (Beverly, MA, USA)
Atg7 (D12B11)	#8558	Cell Signaling Technology (Beverly, MA, USA)
Atg5 (D5F5U)	#12994	Cell Signaling Technology (Beverly, MA, USA)
Atg3	#3415	Cell Signaling Technology (Beverly, MA, USA)
Beclin-1 (D40C5)	#3495	Cell Signaling Technology (Beverly, MA, USA)
LC3A/B (D3U4C)	#12741	Cell Signaling Technology (Beverly, MA, USA)
β-actin	#47778	Santa Cruz Biotechnology (Dallas, TX, USA)
Pink1 (C-3)	#518052	Santa Cruz Biotechnology (Dallas, TX, USA)
Parkin (PRK8)	#32282	Santa Cruz Biotechnology (Dallas, TX, USA)
LAMP-1 (H4A3)	#20011	Santa Cruz Biotechnology (Dallas, TX, USA)
Bafilomycin A1 (Baf-1)	HY-100558	MedChemExpress (NJ, USA)
mdivi-1	475856	Sigma-Aldrich (St. Louis, MO, USA)
N-acetylcysteine (NAC)	A9165	Sigma-Aldrich (St. Louis, MO, USA)
WST assay kit	EZ-3000	DoGenBio (Seoul, South Korea)
CellROX™ Green Reagent	C10444	Thermo Fisher Scientific (Waltham, MA, USA)
JC-1 Dye	T3168	Thermo Fisher Scientific (Waltham, MA, USA)
MitoView™ Green	70054	Biotium (Fremont, CA, USA)
MitoTracker™ Red CMXRos	#9082	Cell Signaling Technology (Beverly, MA, USA)
EZ Total RNA Miniprep kit	EP301-50N	Enzynomics (Daejeon, South Korea)
TOPscript RT DryMIX (dT18/dN6)	RT220	Enzynomics (Daejeon, South Korea)
TOPreal SYBR Green qPCR PreMIX	RT500S	Enzynomics (Daejeon, South Korea)
